# Diet quality indices and their associations with health-related outcomes in children and adolescents: an updated systematic review

**DOI:** 10.1186/s12937-020-00632-x

**Published:** 2020-10-24

**Authors:** Phoebe Dalwood, Skye Marshall, Tracy L. Burrows, Ashleigh McIntosh, Clare E. Collins

**Affiliations:** 1 Bond University Nutrition & Dietetics Research Group, Faculty of Health Sciences & Medicine, Bond Universtiy, Robina, Queensland 4226 Australia; 2 Nutrition Research Australia, Sydney, New South Wales Australia; 3 School of Health Sciences, Faculty of Health and Medicine, The University of Newcastle, Callaghan, NSW 2308 Australia; 4 Priority Research Centre in Physical Activity and Nutrition, The University of Newcastle, Callaghan, NSW 2308 Australia

**Keywords:** Diet quality, diet index, pediatrics, Child, Infant, Adolescent, Nutrition assessment, Child development, Non-communicable diseases, Systematic review

## Abstract

**Background:**

To describe a-priori diet quality indices used in children and adolescents, appraise the validity and reliability of these indices, and synthesise evidence on the relationship between diet quality and physical and mental health, and growth-related outcomes.

**Methods:**

Five electronic databases were searched until January 2019. An a-priori diet quality index was included if it applied a scoring structure to rate child or adolescent (aged 0–18-years) dietary intakes relative to dietary or nutrient guidelines. Diagnostic accuracy studies and prospective cohort studies reporting health outcomes were appraised using the Academy of Nutrition and Dietetics Quality Criteria Checklist.

**Results:**

From 15,577 records screened, 128 unique paediatric diet quality indices were identified from 33 countries. Half of the indices’ scores rated both food and nutrient intakes (*n* = 65 indices). Some indices were age specific: infant (< 24-months; *n* = 8 indices), child (2–12-years; *n* = 16), adolescent (13–18 years; *n* = 8), and child/adolescent (*n* = 14). Thirty-seven indices evaluated for validity and/or reliability. Eleven of the 15 indices which investigated associations with prospective health outcomes reported significant results, such as improved IQ, quality of life, blood pressure, body composition, and prevalence of metabolic syndrome.

**Conclusions:**

Research utilising diet quality indices in paediatric populations is rapidly expanding internationally. However, few indices have been evaluated for validity, reliability, or association with health outcomes. Further research is needed to determine the validity, reliability, and association with health of frequently utilised diet quality indices to ensure data generated by an index is useful, applicable, and relevant.

**Registration:**

PROSPERO number: CRD42018107630.

## Background

The prevalence of non-communicable diseases (NCDs) including type 2 diabetes mellitus (T2DM), cardiovascular disease (CVD), and chronic respiratory disease experienced by children and adolescents aged 0 to 18-years is increasing [1, 2]. Four hundred new cases of T2DM are diagnosed annually in Australians aged 10–24-years [3]. Hypertension, a risk factor of CVD, is present in 6–7% of children and adolescents in Australia, the United Kingdom, and the United States of America (USA) [4–6]. Of concern, NCDs adversely affect growth, development, and maturation in childhood and adolescence [7], leading to compromised adult health and reduced life expectancy [8]. Hence, the prevention of NCDs in childhood is a global priority, requiring a multi-pronged approach to address major NCD risk factors [9]. These risk factors include diet quality, healthcare access, and substance abuse, which affect physical growth and mental development [10], with poor diet quality identified as one of the largest contributors to the global burden of NCDs [11].

Diet quality is broadly defined as a dietary pattern or an indicator of variety across key food groups relative to those recommended in dietary guidelines [12]. High diet quality thereby reflects achieving more optimal nutrient intake profiles and a lower risk of diet-related NCDs [13]. Diet quality can be influenced by confounding factors, including cultural and food environment, socio-economic status, child and family food preferences, and nutrition recommendations relevant to age, sex, country, and/or culture of the individual [14]. Diet Quality Indices (DQIs) are assessment tools that can be used to quantify the overall quality of an individual’s dietary intake by scoring food and/or nutrient intakes, and sometimes lifestyle factors, according to how closely they align with dietary guidelines [12]. There are a variety of DQIs which utilise a range of scoring matrices. Some use frequency of food or food group consumption, others use nutrient intakes which require estimation prior to scoring, and some include both.

Due to the link between dietary intake in childhood and NCDs in both childhood and adulthood, the accurate measurement of paediatric diet quality is essential both to understand current intakes as well as evaluate the effect of interventions [15, 16]. Reflecting this need, the use of DQIs is increasing not only in research and epidemiology, but also in community health and clinical settings where DQIs may form part of dietary education and self-monitoring interventions [14, 17–20]. A systematic review of paediatric DQIs which included papers published up until October 2013 identified 80 individual DQIs used in paediatric population samples, some of which identified cross-sectional associations with growth and health outcomes such as body weight, early onset puberty, and blood pressure [14].

Given the increasing number of DQIs identified in the previous review used or created for research, the diversity in the tools, and the different settings, age groups, and countries they are used amongst, there is a need to update the previous systematic review to identify valid DQIs and their associations with health outcomes [14]. Therefore, the aims of this systematic review update are to; 1) summarise a-priori DQIs used in child and adolescents; 2) appraise the validity and reliability of paediatric diet quality indices; and 3) synthesise the evidence on the relationship between diet quality and physical health, mental health, and growth-related outcomes among paediatric samples.

## Methods

### Study design

A systematic literature review was conducted and reported according to the Preferred Reporting Items for Systematic Reviews and Meta-Analyses (PRISMA) guidelines [21] and registered prospectively with the International Prospective Register of Systematic Reviews (PROSPERO number: CRD42018107630).

### Search strategy

The search was designed as an update of the 2014 systematic review [14]. Medline (PubMed) and CINAHL were searched from 31 October 2013 to 11 January 2019. To broaden the search, the current review also searched Embase, Web of Science, and CENTRAL from database inception to 11 January 2019. The strategy used both controlled-vocabulary and keywords, and was designed for PubMed and translated for use in other databases using Polyglot Search Translator [22]. The translated search strategies were checked for accuracy by a librarian, and two authors (PD and SM), then further adapted for each database after examination of sensitivity and specificity by using a target of one eligible study per 100 records retrieved, with an estimated 150 eligible studies (Appendix). To support the systematic search update, snowball searching of reference lists of identified papers was conducted and the previous review [14] was examined to include any eligible studies the current search strategy didn’t identify.

### Eligibility criteria

Table 1 describes the eligibility criteria used to identify studies to answer the research questions; a study was included if it addressed one or more of the research questions. Studies published in English and Mandarin (translated to English by colleagues) were included. Studies published in other languages were included if they could be translated using Google translate [23]. For this review, a DQI was defined as any assessment tool which applied a quantitative score to food (i.e., frequency of consumption) or nutrient intake, where the scoring system reflected pre-defined national dietary or nutrient guideline/s (i.e., the DQI scoring system was developed a-priori). Diversity and variety indices that score or count the variety of foods consumed without regards to a dietary standard were excluded. Excluded lifestyle indices were any scoring system which had ≥2 scoring components on behaviours such as exercise, sedentary activities, or smoking.

**Table 1:** Eligibility criteria of original studies included in this review according to the population, indicator, comparator, outcomes, and study design (PICOS) format.

	Inclusion criteria	Exclusion criteria
Population	children and adolescents aged 0–18 years old or sample mean age of ≤18 years old	DQI applied to household or menu
Indicator^a^	1) Reported the development of an a-priori DQI, 2) Assessed the validity or reliability of an a-priori DQI, and/or 3) Reported prospective health-outcomes according to an a-priori DQI	DQI reflecting only part of a guideline (e.g. fruit/vegetables only), DQI was not a-priori (e.g. diet diversity scores or food variety scores which do not score according to a pre-established diet or nutrient guideline), or lifestyle indices.
Comparator	Not applicable	Not applicable
Outcomes^a^	1) Scoring structure and characteristics 2) Concurrent, predictive^b^, or content validity; inter-rater reliability 3) Physical health, mental health, or growth-related outcomes	Physical health, mental health, or growth-related outcomes measured cross-sectionally
Study design^a^	1) Any original research study design 2) Diagnostic accuracy studies 3) Prospective observational studies^b^	Review studies, abstracts, and non-peer reviewed papers.

^a^Indicator, Outcomes, and Study design are different for each research question (1, 2, and 3 respectively).

^b^It should be acknowledged that there is overlap between these two eligibility criteria. Prospective health outcomes are frequently used as a measure of predictive validity. Any instance where prospective health outcomes were examined for the purposes of evaluating predictive validity was eligible for inclusion in aim 2 and included in this study as assessing DQI validity

### Study selection and data extraction

Identified records were de-duplicated using Systematic Review Assistant-Deduplication [24] followed by a manual search in Endnote [25]. Titles and abstracts of papers were screened independently to assess their potential eligibility by two researchers (PD and SM) using Covidence [26], which further removed duplicates. The full texts of potentially eligible records were acquired and screened for eligibility by two researchers independently (PD and SM), with disagreements managed by consensus. Data were extracted from included papers by one researcher (PD) into three standardised tables; with random quality checks by a second researcher SM). For studies which measured prospective health-related outcomes, data were reported in their standard international units at baseline and follow-up, as well as mean change over time where possible.

### Health-related outcomes

Any prospective outcome related to physical health, mental health, or growth was included if the variable was reported relative to DQI score or categories. Health-related outcomes used to describe the sample, but not linked to a DQI score were not considered. Health-related outcomes in adults were considered if they were related to a DQI assessment when the sample was aged < 18 years. In order to assess the ability of the DQI to predict health-related outcomes, outcomes were considered from 1-week after the DQI assessment with no further restriction on timeframe of follow-up. Health-related outcomes reported as the result of an intervention study were not considered as outcomes are likely to reflect the intervention rather than baseline diet quality.

### Study quality

Any study which reported on the validity of a paediatric DQI or health-related outcomes was critically appraised using The Academy of Nutrition and Dietetics Quality Criteria Checklist (QCC) [27], independently by two authors (PD, SM, TB, or CC). Studies which reported the use of a DQI but didn’t report validity, reliability, or health-related outcomes were not critically appraised as study quality was not relevant to research question 1. Any disagreements in study quality were settled by consensus. The Academy QCC is a critical appraisal tool suitable to evaluate the risk of bias for any study design, including diagnostic, intervention, or observational. The QCC rates the quality of the study as positive, negative, or neutral reflecting risk of bias in participant selection, generalisability, data collection, and analysis [27]. Studies found to have negative study quality were not excluded.

## Results

**Fig. 1:**
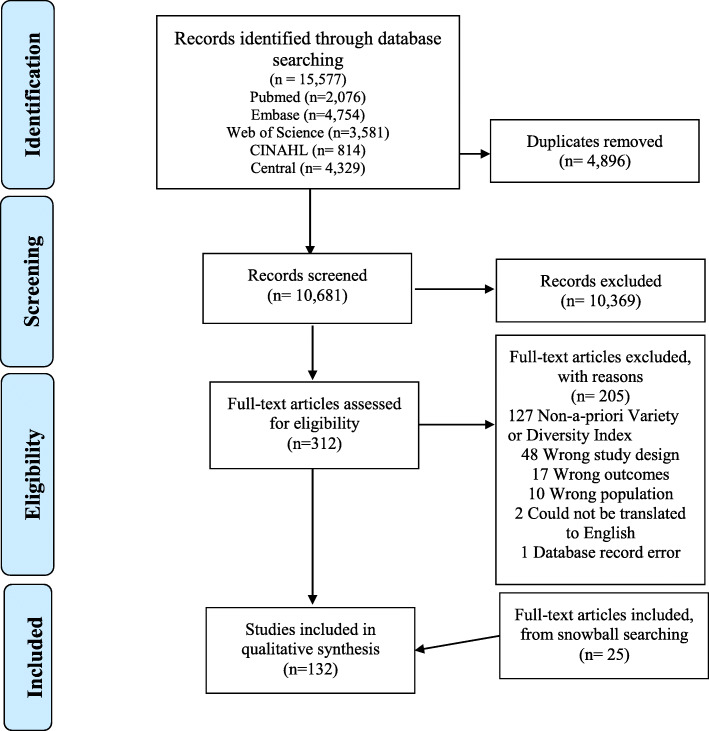
PRISMA flow diagram demonstrating selection of studies.

Of 15,577 records identified in the search, 4896 were duplicates. After title and abstract screening, 312 full texts were assessed against the eligibility criteria, with 132 papers included, including 22 identified through snowball searching (Fig. 1). The main reasons for exclusion were use of a non-a-priori diversity or variety index (*n* = 127), study design (*n* = 48), or study outcomes (*n* = 48). From the 132 included studies, 81 diet quality indices were identified by the current search strategy in addition to those identified in the original systematic review [14]. Of the 80 indices described in the original review [14], 47 were eligible in the current review update and were primarily identified from the current search strategy but was supported by the snowball search (Fig. 1), leading to a combined total of 128 unique indices designed for and/or used among children and adolescents. Of these, 39 included papers had evaluated the validity and/or reliability of 37 DQIs, while 12 evaluated the association of 12 DQIs with prospective health outcomes.

### Characteristics of diet quality indices developed for or used in paediatric samples

The 128 DQIs were developed across 33 countries, with most being designed for the USA (*n* = 23), Australia (*n* = 16), Germany (*n* = 11), and Brazil (*n* = 8) (Table 2). There were 23 DQIs created outside of the USA such as Australia, Belgium, Canada, and Gaza with scoring methods based on the Dietary Guidelines for Americans (Table 2). Very few indices were identified in developing countries (*n* = 7) [262]. Those identified were from India, Indonesia, and Guatemala [134, 138, 141] and were typically brief tools more appropriate for field work, assessing frequency of consumption or dietary patterns and used dietary guidelines from other countries such as the USA to assess diet quality [134, 138, 141]. Thirteen (10%) DQIs were adaptations of the Diet Quality Index (DQI) [250], and 22 (17%) were adaptations of the Health Eating Index (HEI) [227]. These adaptions reflected changes to the scoring system to be more applicable to different countries or age groups. Four identified DQIs were designed for adults and subsequently used among children and adolescents without being adapted [89, 106, 127, 250].

**Table 2:** Description and purpose of diet quality indices which have been designed for use or used in paediatric populations presented alphabetically by country (*n* = 128 indices)

Index & original author	Type of index	Country of origin	Dietary assessment method	Purpose	Scoring	Age group designed for	Validated^a^
Menzies remote short-item dietary assessment tool (MRSDAT) [derives DGI-CA score]; Tonkin et al. (2018) [28]	Food	Australia (Remote Aboriginal Communities)	1) 24-h recalls	Reflects adherence to Australian Dietary Guidelines [29] and traditional food consumption	8 components, (6 food groups + breast feeding & consumption of traditional foods). Scoring not described	Young children (6-36 m)	Yes
Menzies remote short-item dietary assessment tool (MRSDAT) [derives DGI-CA score]; Rohit et al. (2018) [30]						Young children (2-4y)	Yes
Dietary Risk Score (DRS); Bell et al. (2014) [31]	Food	Australia	1) TDQ^b^	Adherence of Toddlers dietary patterns to the Australian Dietary Guidelines [29]	3 components, scored 0–336 (then converted to 0–100), calculated proportionally on intake/ recommendation	Young children (12-36 m)	Yes
Australian Recommended Food Score for Preschoolers (ARFS-P); Burrows et al. (2014) [19]	Food & nutrient	Australia	1) FFQ - Australian Eating Survey Pre-schooler Version (AES-P)	Reflects dietary variety within recommended food groups from the Australian Dietary Guidelines [29]	8 components, scored 0–73, points awarded & summed	Young children (2-5y)	Yes
Healthy Preference Index (HPI); Russell et al. (2007) [32]	Food	Australia	1) Food preferences & appetite traits questionnaire	Reflects food preferences & adherence to the Australian Guide to Healthy Eating for children & adolescents [29]	10 components, scored 1–100, points awarded & summed.	2–5	No
Australian Child & Adolescent Recommended Food Score (ACARFS); Marshall et al. (2012) [33]	Food	Australia	1) FFQ	Reflects adherence to the 2003 Australian Dietary Guidelines for Children & Adolescents [29]	8 components, scored 0–73, points awarded & summed	6-14y	No
Unnamed healthy dietary score; Gasser et al. (2017) [34]	Food	Australia	1) 24-h recall	Reflect adherence to the 2013 Australian Dietary Guidelines [29]	7 components, scored 0–14, points awarded & summed	Not specified	No
Dietary Guideline Index for Children & Adolescents (DGI-CA); Golley et al. (2011) [35]	Food	Australia	1) 24 h recall	Reflects adherence to the 2003 Australian Dietary Guidelines for Children & Adolescents & 1998 Australian Guide to Healthy Eating [29]	11 components, scored 0–100, calculation by nutrient analysis.	Not specified	Yes
Dietary Guideline Index (DGI); Lioret et al. (2014) [36]	Food & nutrient	Australia	1) FFQ	Reflecting adherence to the 2003 Australian Dietary Guidelines for Children and Adolescents [29]	10 components, scored 0–10, points awarded and summed for a total score of 0–100.	Not specified	No
Healthy and Unhealthy Diet score, Jacka et al. (2010) [37]	Food	Australia	1) 14-item dietary questionnaire	Adherence to Dietary Guidelines for Children and Adolescents in Australia [29]	Healthy diet core: 4 components, scored 0–4, points awarded and summed. Unhealthy diet score: 4 components, scored 5–30, points awarded and summed	Not specified	No
Raine Eating Assessment in Toddlers (EAT) score; Meyerkort et al. (2012) [38]	Food & nutrient	Australia	1) 24-h recalls 2) FFQ	Reflects adherence to the Dietary Guidelines for Children and Adolescents in Australia [29]	7 components (5 healthy, 2 unhealthy), scored 0–70 points awarded & summed	Not specified	No
The Diet Score; Nyaradi et al. (2015) [39]	Food & nutrient	Australia	1) Modified 24-h dietary recall	Reflects adherence to the Dietary Guidelines for Children and Adolescents in Australia [29]	7 components (healthy and unhealthy), scored 0–70, points awarded & summed	Not specified	No
Unnamed Diet Quality Index; Li et al. (2012) [40]	Food & nutrient	Australia	1) FFQ	Reflects adherence to the Australian Guide to Healthy Eating [29] and Nutrient Reference Values for Australia & NZ [41]	15 components, scored 20–150, calculated by nutrient analysis & servings.	Not specified	No
Core Food Variety Score (CFVS); Scott et al. (2012) [42]	Food	Australia	1) 24 h recall	Reflects adherence to the Australian Guide to Healthy Eating [29]	6 food groups, scored 0–34, points awarded & summed	Not specified	No
Fruit and Vegetable Variety Score (FVVS); Scott et al. (2012) [42]	Food	Australia	1) 24 h recall	Reflects adherence to the Australian Guide to Healthy Eating [29]	6 food groups, scored 0–16, points awarded & summed	Not specified	No
Obesity Protective Dietary Index (OPDI); Spence et al. (2013) [43]	Food	Australia	1) 24 h recall	Reflects adherence to a non-obesogenic diet (with non-core food groups) based on dietary guidelines for Americans [44]	3 food groups, scored 0–30, based on quantile ranking & summed	Not specified	Yes
Short Food Frequency Questionnaire Diet Quality Index (sFFQ-DQI); Kunaratnam et al. (2018) [45]	Food & nutrient	Australia	1) short food frequency questionnaire (sFFQ) 2) 3-day food records (3d-FR)	Reflects adherence to the 2013 Australian dietary guidelines for children and adolescents [29]	13 components, scored 0–5, points awarded and summed, for a total score of 0–65.	2-5y	Yes
Diet Quality Index for Preschool Children (DQI-CH); (Huybrechts et al. (2010) [46]	Food & behaviour	Belgium	1) Food diary/record 2) FFQ	Reflects compliance with Flemish Food-Based Dietary Guidelines [47]	4 components, scored −25 - 100 calculated as percentage of food group intakes	2 – 6y	Yes
Daily Diversity Index (DDI); Sabbe et al. (2008) [48]	Food	Belgium	1) FFQ	Reflects adherence to the 2000 American Food Guide Pyramid [49]	5 components, scored 0–5, points awarded & summed	Not specified	No
Healthy Eating Index for Brazilians (HEI); Rauber et al. (2014) [50]	Food & nutrients	Brazil	1) 24-h dietary recall	Reflects adherence to US Department of Agriculture dietary guidelines [44] with some modifications to meet recommendations of the Brazilian dietary guidelines [51]	10 components, scored 0–100, calculated based on adherence to dietary guidelines, points awarded & summed	Children (3-8y)	Yes
Índice de Alimentação do Escolar (ALES – School Child Diet Index); Molina et al. (2010) [52]	Food	Brazil	1) FFQ	Reflects adherence to recommended national dietary guidelines [51]	15 components, scored −1, 0 or 1, points awarded and summed for a total score of −9-14.	Not specified	No
Adapted Healthy Eating Index (adHEI) Conceicao et al. (2018) [53]	Food & nutrient	Brazil	1) 24-h dietary recall	Reflects adherence to dietary guidelines for Brazilian children [51]	10 components, scored 0–10, points awarded and summed.	Not specified	No
Brazilian Healthy Eating Index (BHEI) [Índice de Qualidade da dieta (IQD)], Fisberg et al. (2004) [54]	Food and nutrients	Brazil	1) 24-h dietary recall	Adherence to The Adapted Food Pyramid for Brazilians [55]	10 components, scored 0–10, points awarded & summed for a total score of 0–100	Not specified	Yes
The Revised Brazilian Healthy Eating Index (BHEI-R), Previdelli et al. (2011) [56]	Food and nutrients	Brazil	1) 24-h dietary recall	Reflects adherence to the Brazilian dietary guidelines [51] recommendations and the measurement of dietary risk factors for chronic diseases	12 components, scored 0–5, 0–10 or 0–20 for the % of total energy value, with all components summed	Not specified	No
Brazilian food habits (BHEI-R); Rodrigues et al. (2016) [57]	Food & nutrients	Brazil	1) FFQ 2) 24-h recalls	Reflects adherence to the Brazilian dietary guideline’s recommendations [51]	12 components, scored 0–5, 0–10 or 0–20, point awarded and summed.	Not specified	No
The Diet Quality Index associated with the Digital Food Guide (DQI-DFG) or “Índice de Qualidade da Dieta associado ao Guia Alimentar Developed by Caivano et al. (2013) [58] in adults, Baldasso et al. (2016) [59] used in adolescents. Digital” (IQD-GAD)	Food & nutrients	Brazil	1) 24-h recall 2) Food records	Reflects adherence to US healthy eating guidelines, dietary reference intakes and food pyramid [44]	12 components, 1–4 based on moderation, 5–12 based on adequacy, scored 0–100, points awarded and summed.	Not specified	No, Validated in adults (Caivano et al. 2013)
Revised Diet Quality Index (IQD-R); Wendpap et al. (2014) [60]	Food & nutrients	Brazil	1) FFQ	Reflects adherence to recommendations of the 2006 Food Guide for the Brazilian Population [51]	11 components, scored 0–100, calculated by nutrient analysis & servings	Not specified	No
Canadian Healthy Eating Index (HEI-C-2010); Jessri et al. (2017) [61] developed in adults, Nshimyumukiza et al. (2018) [62] used among children	Food & nutrient	Canada	1) 24-h recalls	Reflects adherence of adequacy and moderation to the 2007 Canada’s Food Guide [63]	11 components, scored 0–100, calculated by nutrient analysis & servings	≥ 2y	No, Validated In adults (Jessri et al. 2017)
Canadian Healthy Eating Index (HEI-C); Glanville et al. (2006) [64]	Food & nutrient	Canada	1) 24 h recall 2) FFQ	Reflects adherence to the 1993 Canada’s Good Guide to Healthy Eating & 1990 Canadian Nutrient Recommendations [65]	9 components, scored 0–100, calculated by nutrient analysis & servings.	≥3y	No
Canadian Health Eating Index-2009 (HEIC-2009)^;^ Woodruff et al. (2010) [66]	Food & nutrient	Canada	1) 24-h recall 2) FFQ	Reflects adherence to the 2007 Eating Well with Canada’s Food Guide [63]	9 components, scored 0–100, calculated by nutrient analysis & servings	≥3y	No
Canadian Healthy Eating Index (HEI-C); Wang et al. (2015) [67]	Food & nutrient	Canada	1) 24-h recalls	Reflects adherence to the 2007 Eating Well with Canada’s Food Guide [63]	9 components, scored 0–100, calculated by nutrient analysis & servings	9-13y	No
School Heathy Eating Index (School-HEI), Tugault-Lafleur et al. (2017) [68]	Food & nutrient	Canada	1) computer-assisted 24-h dietary recall	Dietary compliance with Canadian dietary guidance from the 2007 version of Canada’s Food Guide [63]	2 subsections; scored 0–100, Adequacy: 8 components, scored 0–60. Moderation; 3 components scored 0–40, points awarded & summed	Not specified	No
Unnamed diet quality index; Absolon et al. (1988) [69]	Food & nutrient	Canada	1) 24-h dietary recall	Adherence to Canada’s Food Guide and Recommended Nutrient Intakes for Canadians [63]	5 components, scored 0–8, points awarded & summed	Not specified	No
Adapted Youth Healthy Eating Index (aYHEI); Protudjer et al. (2012) [70]	Food & nutrient	Canada	1) FFQ	Assesses dietary adherence to the United States’ Department of Agriculture’s Dietary Guidelines for Americans [44]	10 components, scored 0–85, point awarded & summed	Not specified	No
Chinese Healthy Eating Index (CHEI); Yuan et al. (2017) [71]	Food & nutrient	China	1) 24-h recalls	Reflects adherence to the updated Dietary Guidelines for Chinese (DGC-2016) [72]	17 components, scored 0–100, calculated by nutrient analysis & servings	≥2y	No, Validated among adults, Yuan et al. (2018)
The Chinese Children Dietary Index (CCDI); Cheng et al. (2016) [73]	Food, nutrient & behaviour	China	1) 24-h recalls	Reflects adherence to Chinese Dietary Guidelines and Dietary Reference Intakes [72]	16 components, scored 0–160, calculated based on intake/ recommendations points awarded & summed	Not specified	Yes
Healthy nutrition score based on food intake for pre-schoolers (HNSP), Peng et al. (2015) [74]	Food & nutrient	China	1) 24-h recalls 2) 3-day food records	Adherence to Chinese Dietary Guidelines [72] for preschool children to detect vitamin A deficiencies	10 components, scored 0–100, points awarded and summed	Not specified	Yes
Foods E-KINDEX; Lazarou et al. (2009) [75]	Food & behaviour	Cyprus	1) FFQ	Reflects risk of being overweight or obese based on adherence to the Mediterranean dietary pattern [76]	13 components, scored 0–37, unspecified scoring method	Not specified	No, (previously validated in children)
Adapted diet quality index; Knudsen et al. (2012) [77] developed in adults, Rohde et al. (2016) [78] used in children	Food	Denmark	1) 4-d diet record	Compliance of children’s diet with the Danish national guidelines [79]	6 components, scored 0–6, calculated as a ratio of reported intake to recommended intake.	Not specified	No
Complementary Feeding Utility Index (CFUI); Golley et al. (2012) [80]	Food & behaviour	England	1) FFQ & 2) independent questionnaire	Reflects adherence to complementary feeding guidelines in Australia [81], NZ [82] USA [83] and UK [84]	14 components, scored 0–1, calculated by summing of probability functions	Not specified	No
Unnamed Diet Quality Score (DQI); Okubo et al. (2015) [85]	Food & nutrient	England	1) FFQ	Describe compliance with the Japanese Spinning Top Guide [86]	Calculated using reported intake & recommendations results split into tertiles, scored 0–8.	Not specified	No
NutricheQ Tool, Rice et al. (2015) [87]	Food	European countries^e^	1) Food diary/record	Based on suggestions from family paediatricians, the user-requirements of European nutrition experts, and evidence from the literature [87]	3 components, each of the 18 questions has a minimum of 0 and maximum of 3 points, points awarded & summed	Young children (≥12 m)	Yes
The Diet Quality Index for Adolescents (DQI-A); Vyncke et al. (2013) [88]	Food	European countries^h^	1) 24-h recalls	Reflects adherence to Adolescent Flemish food based dietary guidelines [47]	3 components, dietary diversity, quality and equilibrium calculated.	Adolescents (12.5–17.5y)	Yes
Healthy Diet Indicator (HDI); Huijbregts et al. (1997) [89]	Food & nutrient	European counties^c^	1) Diet history 2) food diary/record	Reflects WHO guidelines to prevent chronic disease [90]	9 components, scored 0–9, points awarded & summed	Not specified	No
Healthy Dietary Adherence Score (HDAS); Arvidsson et al. (2017) [91]	Food	European countries^d^	1) FFQ	Reflect adherence to healthy dietary guidelines common for all eight countries participating in the IDEFICS study [92]	5 components, scored 0–10, points awarded & summed	Not specified	No
The Healthy Plate Variety Score (HPVS), Oliveira et al. (2015) [93]. Also referred to Healthy Food Variety Index (HFVI) by Jones et al. (2015) [94]	Food	European countries^f^	1) FFQ	Reflects adherence to the US Health Eating Guidelines recommendations and variety [44]	5 components, scored 0–1, points calculated and summed.	Not specified	No
The Diet Quality Index for Adolescents (DQI-A), De Vriendt et al. (2012) [95]	Food	European countries^g^	1) 2-day dietary recall	Reflecting dietary diversity, quality and equilibrium related to Flemish dietary guidelines [96]	Dietary Diversity: The extent of food groups consumed, scoring not described. Dietary Quality: 3 components, each food item scored: 1, 0, −1, points awarded, summed and divided by the number of foods consumed. Dietary Equilibrium: Indicates adherence to portion sizes, scoring not described. Scores were summed for overall DQI-A score from −33·3 and 100%.	Not specified.	No
Ideal Diet Score, also referred to as Healthy Diet Score, Lloyd-Jones et al. (2010) [97] developed in adults, Henriksson et al. (2017) [98] used among chidren	Food & nutrient	European countries^i^	1) 24-h recalls	Adherence to American Heart Association guidelines for primary prevention of atherosclerotic cardiovascular disease beginning in childhood [99]	5 components, points awarded based on cut-off values and summed for a total score of 0–5.	Not specified	No
Children’s Index of Diet Quality (CIDQ); Röytiö et al. (2015) [100]	Food & nutrient	Finland	1) Food Consumption Questionnaire 2) 7-d food recall	Reflects adherence to Finnish nutrition recommendations, based on the Nordic nutrition recommendations [101]	14 components, calculated intake compared to recommendations, scored 0–21, points awarded & summed.	Children (2-6y)	Yes
Baltic Sea Diet Score (BSDS); Kanerva et al. (2013) [102] developed in adults, Haapala et al. (2017) [103] used in children	Food	Finland	1) 4-day food record	Assess dietary pattern reflecting the Baltic Sea Diet Pyramid [101]	8 components, calculated ratio of reported intake to recommended intake, points awarded.	Not specified	No
Finnish Children Healthy Eating Index (FCHEI); Kyttälä et al. (2014) [104]	Food & nutrient	Finland	1) 3-d food records	Reflects adherence to the Nordic dietary guidelines [101]	5 components, scored according to food groups and ages, points awarded and summed for a total score of 0–34/41/42 depending on age.	Not specified	Yes
Dietary Adequacy Score (DAS); Guthrie et al. (1981) [105]	Food	Unknown	1) 24 h recall; 2) FFQ	Reflects adequacy of (unknown) Recommended Dietary Allowances for an unknown population	4 components, scored 0–16, points awarded & summed	Not specified	No
Dietary Adequacy Score (DAS); Shatenstein et al. (1996) [106]		Gaza	1) FFQ	Reflects adequacy of Recommended American Dietary Guidelines [44]			
PANDiet score; Developed by Verger et al. (2012) among ≥18y, Verger et al. (2016) [107]	Food & nutrient	UK	1) 24-h recal	Reflects adequate nutrient intake based on UK dietary guidelines [108]	2 components (adequacy and moderation) are calculated, and the mean of the two sub-scores are then calculated ranging from 0 to 100.	Infants & young children (12-18 m)	Yes
PANDiet score; Schoen et al. (2017) [109]	Food & nutrient	Germany	1) 3-d weighed food record	Reflects adherence to national reference values for nutrient intake in children based on UK dietary reference values [110].		Infants & young children (9-24 m)	No
Unnamed Dietary Quality Index (DQI), Alexy et al. (2003) [111]	Nutrient	Germany	1) Dietary survey including 3d weighed dietary record.	Reflects adherence to the Dietary Guidelines for an OMD for Children & Adolescents and comparison to reference values for nutrient intakes [112]	12 components, scored 0–12, points awarded & summed	≥2y	Yes
Healthy Nutrition Score for Kids & Youth (HuSKY); Kleiser et al. (2009) [113]	Food	Germany	1) FFQ	Reflects adherence to the Dietary Guidelines for an OMD for Children & Adolescents [112]	11 components, scored 0–100, calculated as ratio of food group intakes	3–17y	No
Recommended Food Group Change Score (RFS); Alexy et al. (1999) [114]	Food	Germany	1) Food diary/ record	Reflects average change in amounts of deviation from the OMD food groups based on individualised recommendations [112]	Number of components change depending on individualised recommendations, scored as a negative or positive percentage of change, servings summed	Not specified	No
Total Food Group Change Score (TFS); Alexy et al. (1999) [114]	Food	Germany	1) Food diary/ record	Reflects average change in amounts of deviation from all OMD food groups [112]	11 components, scored as a negative or positive percentage of change, servings summed	Not specified	No
Nutrient Improvement Score (NIS); Alexy et al. (1999) [114]	Nutrient	Germany	1) Food diary/ record	Reflects average change in dietary intake of German reference values for nutrient intakes [112]	16 components, scored as a ‘-‘or ‘+’ percentage of change, scored by nutrient analysis	Not specified	No
Nutrition Quality Index (NQI) Gedrich et al. (2001) [115]	Nutrient	Germany	1) Food diary/ record	Reflects adequacy as compared the 2002 German, Austrian & Swiss Dietary Reference Values [116]	13–17 components, scored 0–100, calculated by nutrient analysis	Not specified	No, Validated in adults
Nutrition Quality Index (NQI), Cheng et al. (2010) [117]	Nutrient	Germany	1) 3-d weighed food diary/record	Reflects the extent to which a child meets the nutritional recommendation for particular nutrients [118], and indicated diet quality compared to dietary reference values from the German and Austrian Nutrition Society [119].	Components scored 0–100, calculated by nutrient analysis.	Not specified	No
Diet Quality Score; Kohlboeck et al. (2012) [120]	Food	Germany	1) FFQ	Reflects adherence to the OMD food groups based [112]	11 components, scored 0–11, points awarded & summed	Not specified	No
The Preschoolers Diet–Lifestyle Index (PDL-index); Manios et al. (2010a) [121]	Food & behaviour	Greece	1) 24-h dietary recalls 2) Weighed food records) Or Food diaries	Reflects adherence to American Food Guide Pyramid [49]& Canadian Food Guide [65]	11 components, scored 0–44, points awarded & summed	2-5y	Yes
Food Index (FI); Magriplis et al. (2015) [122]	Food	Greece	1) FFQ	Reflects adherence to food recommendations-dietary guidelines (USDA data [123] and the Mediterranean Food Pyramid guidelines [76]).	2 sections: 6 obesogenic and 8 non-obesogenic components. 14 components scored 16–64, calculated with a weighting of 1 or 1.5 for non-obesogenic foods	9-13y	Yes
Unhealthy Food Choices Score (UFCS); Yannakoulia et al. (2004) [124]	Food	Greece	1) FFQ	Reflects adherence to a number of Greek [125]& US Dietary Guidelines [126]	9 categories, scored 9–45, negative & positive points awarded & summed	11–15y	No
Mediterranean Diet Score (MDS); Trichopoulou et al. 1995 [127]	Food & nutrient	Greece	1) FFQ, 2) food diary/record	Reflects adherence to the Mediterranean dietary pattern [76]	8 components, scored 0–8, points awarded & summed	Not specified	No
E-KINDEX; Lazarou et al. (2008) [128]	Food & behaviour	Greece	Unclear	Reflects risk of being overweight or obese from the CYKIDS study [129]	Composed of 3 indices with a total of 30 components, scored 1–87	Not specified	No
Healthy Lifestyle–Diet Index (HLD-Index); Manios et al. (2010b) [130]	Food	Greece	1) 24-h dietary recalls	Adherence to guidelines reflecting Mediterranean dietary patterns [131] and US Department of Agriculture (USDA)‘s My Pyramid [49]	10 components, each scored 0–4, points awarded and summed with a total score of 0–40.	Not specified	Yes
Revised Healthy Lifestyle Index (R-HLD-Index); Manios et al. (2015) [132]	Food	Greece	1) 24-h dietary recalls	Adherence to the updated dietary recommendations for children proposed by the USDA’s ‘Choose My Plate’ [133]	12 components, each scored 0–4, points awarded and summed with a total score of 0–40.	Not specified	No
USAID Dietary Diversity Score (DDS); Enneman et al. (2009) [134]	Food	Guatemala	1) 24 h recall	Indicates diet diversity, quality and quantity of complementary foods based on a publication of USAID (US Agency for International Development) [135] and Guatemalan dietary guide [136]	8 components, scored 0–8, servings summed	Not specified	No
Cooking Pot Dietary Diversity Score (DDS); Enneman et al. (2009) [134]	Food	Guatemala	1) 24 h recall	Indicates diet diversity in adherence with the Guatemalan dietary guide translated [136]	6 components, scored 0–6, servings summed	Not specified	No
INCAP Papers Dietary Diversity Score (DDS); Enneman et al. (2009) [134]	Food	Guatemala	1) 24 h recall	Indicates diet diversity in adherence with INCAP protocol [137]	25 components, scored 0–25, servings summed	Not specified	No
Adolescent Micronutrient Quality Index (AMQI); Chiplonkar et al. (2010) [138]	Food	India	1) 24 h recall	Reflects adherence to the 2005 Dietary Guidelines for Indians [139] & the 2005 Dietary Guidelines for Americans [140]	13 components, scored 0–100, points awarded & summed, unspecified scoring method	Not specified	No
Expected Food Pattern (PPH) also called Desirable Dietary Pattern (DDP) score; Prasetyo et al. (2013) [141]	Food & nutrient	Indonesia	1) 24-h recall	Dietary quality indicator Reflects adequacy and diversity of diet compared to Indonesian recommendations [142]	9 components, scored 1–30. Calculated from reported intake compared to recommendations, points awarded & summed	Not specified	No
Dietary Approaches to Stop Hypertension (DASH)-style diet score; Fung et al. (2008) [143] developed in women; Asghari et al. (2016) [144] used in children	Food and nutrients	Iran	1) FFQ	Reflects foods and nutrients emphasized or minimised in DASH dietary pattern [145]	8 components, scored 8–40, points awarded and summed.	Not specified	No
Nutrient Adequacy Ratio (NARs), Rouhani et al. (2012) [146]	Nutrient	Iran	1) FFQ	Reflects adherence to dietary recommended intake [147]	10 components, divide daily reported intake by recommended intake (DRI) for each nutrient.	Not specified	No
Mean Adequacy Ratio (MAR), Azadbakht (2014) [148]	Nutrient	Iran	1) FFQ	Reflects adherence to dietary reference intake [147]	Calculated as the ratio of the sum of NAR to the number of nutrients (n 10)	Not specified	No
Modified Healthy Eating Index ‘mHEI’, Hooshmand et al. (2018) [149]	Food & nutrient	Iran	1) FFQ	Reflects the USDA food guide pyramid and dietary guideline [150]	10 components, scored 0–10, points awarded and summed.	Not specified	No
Modified revised children’s diet quality index (M-RCDQI), Keshani et al. (2018) [151]	Food & nutrient	Iran	1) FFQ	Based on two previous studies [73, 152] Reflects adherence to the 2005 Dietary Guidelines for Americans [140] and recommendations adjusted to better reflect the Iranian dietary patterns.	13 components, scored 0–90, points awarded & summed	Not Specified	No
Dietary Guidelines for Americans Adherence Index (DGAI); Fogli-Cawley et al. (2006) [153] developed in adults, Mohseni-Takalloo et al. (2016) [154] used in adolescents.	Food	Iran	1) FFQ	Reflects adherence to the Dietary Guidelines for Americans [140]	20 components, scored 0–20, calculated reported intake/ recommended intake, points awarded and summed.	Not specified.	No
Un-weighted Diet Quality Score (DQS), Perry et al. (2015) [155]	Food & nutrient	Ireland	1) FFQ 2) 24-h recall	Reflects adherence to Irish guidelines and guided by Food Safety Authority of Ireland recommendations [156]	20 components, ‘healthy’ [157] & ‘unhealthy’ [6] components scored −5-25, points awarded & summed	Not Specified	No
Mediterranean diet quality index (M-DQI); Gerber (2006) [158] developed in adults; Tarabusi et al., (2010) [159] Used in children	Food and nutrient	Italy	1) 24-h dietary recall 2) FFQ	Reflects adherence to Dietary recommendations from the 1989 National Academy of Sciences publication [160], adherence to the 1989 Recommended Dietary Allowances [161] with Mediterranean diet adaptions of adolescent’s diets to the Mediterranean diet [162].	7 components, scored 0–14, calculated as a percentage of adherence to the Mediterranean diet, points awarded and summed.	Not specified	No, (validated among adults Gerber, 2016)
Food-based diet quality score; Nishimura et al. (2015) [163] developed in adults; Kuriyama et al. (2016) [164], developed modified score in adolescent/adult;	Food	Japan	1) diet history questionnaire (DHQ)	Assessed the adherence to the food-based Japanese dietary guidelines [86]	6 components, scored 0–10, the score was calculated proportionately between 0 and 10 and summed	18y & adults	No
Korean Dietary Action Guides for Children Adherence Index (KDAGCAI); Choi et al. (2013) [165]	Food & behaviour	Korea	1) FFQ	Reflects adherence to the Korean Dietary Action Guides for Children [166]	19 components, scored 1–5, scores averaged for a total score of 1–5.	Children (3-12y)	No
Dietary Diversity Score (DDS8) & Dietary Diversity Score (DDS8-R); Moursi et al. (2008) [167]	Food	Madagascar	1) 24 h recall	Relation of diet diversity [168] to micronutrient density based on recommended nutrient intakes from FAO/WHO recommendations [169]except for calcium [170]and zinc [171]	8 components, scored 0–8, points awarded & summed	Infants (6-23 m)	No
Dietary Diversity Score (DDS7) & Dietary Diversity Score (DDS7-R); Moursi et al. (2008) [167]					7 components, scored 0–7, points awarded & summed (fats and oils excluded)		
Unnamed Diet quality index for muti-ethnic Asian toddlers, Chen et al. (2018) [172]	Food	Malaysia	1) FFQ	Reflects adherence to the Singapore Dietary Guidelines for toddlers [173]	7 components, scored 0–65, calculated as a ratio of reported intake/ recommended intake, points awarded & summed	Toddlers (1-2y)	Yes
Healthy Eating Index for Malaysians; Lee et al., (2011) [174] developed in adults, Rezali et al. (2015) [175] used in adolescents	Food and nutrient	Malaysia	1) 2-day Dietary Recall	Assessed degree of compliance with recommended Malaysian Dietary Guidelines for Children and Adolescents [176]	9 components, scored 0–10, points awarded and summed and a composite score in percentage was calculated.	Not specified	Yes
Diet quality score for preschool children; Voortman et al. (2016) [177] developed for preschool children, van der Velde et al. (2018) [178] used in school-aged children	Food & nutrient	Netherlands	1) FFQ	Adherence to the dietary recommendations for children from the Dutch Guidelines for a Healthy Diet of 2015 [179]	10 components, scored 0–10, calculated the ratio of reported & recommended intake, ratios summed.	8y	Yes
Diet Quality Score for Preschool Children; Voortman et al. (2015) [177]	Food & nutrient	Netherlands	1) FFQ	Reflects adherence to national and international^j^ guidelines: The Netherlands [180], Germany [181], Switzerland [182] Belgium [183], Northern Ireland [184] and US [185]. Scientific literature on foods that were not consistently reported int these guidelines were also considered (e.g. sugar sweetened beverages, fish and whole milk) [186–188]	10 components, scored 0–10, points awarded and summed	Not specified	Yes
Dietary Index for a Child’s Eating (DICE), Delshad et al. (2018) [189]	Nutrient	New Zealand	1) 4-d estimated food record	Reflects adherence to the recommended NZ Food and Nutrition guidelines [190] and meeting nutrient reference values for Australia and New Zealand [191]	13 components, scored 0–100, points awarded & summed	Children (2-18y)	Yes
Diet Quality Index for NZ adolescents (NZDQI-A); Wong et al. (2013) [192]	Food & Nutrient	New Zealand	1) Food questionnaire 2) 4-day food record	Reflecting adherence of adequacy and variety to the New Zealand Food and Nutrition Guidelines for Healthy Adolescents [193].	5 components, scored 0–100, calculated from adequacy x variety, points awarded & summed	Adolescents (14-18y)	Yes
Healthy Dietary Habits Score for Adolescents (HDHS-A); Wong et al. (2014) [194]	Food & Nutrient	New Zealand	1) 24-h recalls 2) DHQ	Reflecting adherence for NZ Food and Nutrition Guidelines for Healthy Children and Young People [190].	5 components, scored 0–68, points awarded & summed	Adolescents (15-18y)	Yes
Norwegian Adolescent Diet Score; Handeland et al. (2016) [195]	Food & behaviour	Norway	1) FFQ	Reflects adherence to Norwegian dietary recommendations [196]	8 components, scored 0–8, (7 food, 1 physical activity) scored 0 or 1 calculated from cut off values, points summed	Adolescents (14-15y)	Yes
Dietary Diversity Score (DDS); Kennedy et al. (2007) [197]	Food	Philippines	1) 24 h recall, 2) FFQ	Indicates diet diversity in adherence with development & analysis guidelines for developing countries (Arimond et al.*,* 2005; Kennedy & Nantel 2006)	10 components, scored 1–9, servings summed	Not specified	No
Healthy Eating index (HEI); Vilela et al. (2014) [198]	Food & nutrient	Portugal	1) FFQ	Reflects adherence to the WHO food and nutrition policy [199]	7 components, scored 7–28, quartiles calculated for each and scored/reverse scored.	Young children (2y & 4-5y)	No
Diet Quality Index Score (DQIS); Rios et al. (2016) [200]	Food	Puerto Rico	1) FFQ	Reflects adherence to age-specific dietary guidelines by WIC [201], WHO [202] and the American Academy of Paediatrics [203]. (0-24 m not included in national dietary guidelines)	9 components, score 0–55, calculated from adequacy of intake, point awarded & summed.	Infants and toddlers. (0-24 m)	Yes
Diet Quality Score; Crombie et al. (2009) [204]	Food	Scotland	Unclear	Reflects adherence to the Caroline Walker Trust recommendations for under 5y [205]	5 components, dichotomous scoring for each component summed	2-5y	No
Mediterranean dietary pattern (MDP); Trichopoulou et al. (2003) [206] developed in adults, Mariscal-Arcas et al. (2010) [207] used in children - Also referred to as Mediterranean-diet scale	Food & nutrient	Spain	1) FFQ 2) 24-h recall	Reflects the degree of adherence to the traditional Mediterranean diet [76]	9 components, calculated from intakes and recommended intakes	Not specified	No
Mediterranean Diet Quality Index International (Med DQI-I)^;^ Mariscal-Arcas et al. (2007) [208]	Food & nutrient	Spain	1) 24 h recall & FFQ	Reflects worldwide (WHO [209], USA [150, 210] & China [211, 212] adherence to dietary food & nutrient recommendations and included use of Spanish recommended daily intakes [213] with specific Mediterranean adaptations	4 major components each with sub-components, scored 0–100, calculated by nutrient analysis	Not specified	No
Mediterranean Diet Quality Index for children & adolescents (KIDMED); Serra-Majem et al. (2004) [214]	Food & behaviour	Spain	1) 24 h recall & FFQ, 2) independent questionnaire, 3) FFQ & independent questionnaire, 4) 24 h recall	Reflects adherence to the Mediterranean Diet Model [76, 215]	16 components, scored 0–12, points awarded & summed	Not specified	No
Breakfast Quality Index (BQI); Monteagudo et al. (2012) [216]	Food & nutrient	Spain	1) FFQ	Adherence to guidelines of the US [217–219], China [211, 212] Mediterranean dietary patterns [76]	10 components, scored 0–10, points awarded & summed	Not specified	No
Youth Healthy Eating Index-Taiwan (YHEI-TW); Chiang et al. (2011) [220] used, not described; Lee et al. (2012) [221] used in youth.	Food & nutrient	Taiwan	1) 24-h dietary recall 2) FFQ	Reflects adherence to the Dietary Guidelines for Americans [222]	11 components, points awarded and summed for a total score of 0–90.	Not specified	No
Youth Healthy Eating Index-Taiwan Revised (YHEI-TwR-90) Chen et al. (2018) [223]	Food & nutrient	Taiwan	1) 24-h dietary recall 2) FFQ	Reflects adherence to the Dietary Guidelines for Americans [150]	10 components, scores calculated from nutrient analysis and summed, for a total score of 0–90.	Not specified	No
Youth Healthy Eating Index-Taiwan Revised (YHEI-TwR-70); Chen et al. (2018) [223]					8 components, scores calculated from nutrient analysis and summed, for a total score of 0–70.		
Infant & Child Feeding Index (ICFI); Ruel et al. (2002) [224]	Food & behaviour	Designed in the USA for use in Latin America	1) 24 h recall & FFQ, 2) 24 h recall, 3) 7d recall	Reflects adequacy of the 1998 WHO [225]& the 1999 Academy of Educational Development complementary feeding recommendations [226]	5 components, scored 0–12, points awarded & summed	6–36 m	No
Healthy Eating Index (HEI); Kennedy et al. (1995) [227]	Food & nutrient	USA	1) 24 h recall & food diary/ record, 2) FFQ, 3) 24 h recall, 4) Food diary/ record	Reflects adherence to the Dietary Guidelines for Americans & the USDA Food Guide Pyramid (1992) [150]	10 components, scored 0–100, calculated by nutrient analysis	≥2y	Yes
Healthy Eating Index-2010 (HEI-2010); Guenther et al. (2013) [228]	Food & nutrient	USA	1) 24-h recalls	Reflects adherence to the 2010 Dietary Guidelines for Americans [185] and the accompanying USDA Food Patterns [229]	12 components, scored 0–100, calculated by nutrient analysis & servings & summed	≥2y	No, (Later validated by Guenther et al. 2014)
Healthy Eating Index-2005 (HEI-2005); Britten et al. (2006) [230] developed in abstract, not described, Guenther et al. (2008) [231]	Food & nutrient	USA	1) 24 h recall, 2) food diary/record	Reflects adherence to the 2005 dietary guidelines [140] MyPyramid Food Guidance System [232]	12 components, scored 0–100, calculated by nutrient analysis	2–18y	No
Children’s Diet Quality Index (C-DQI); Kranz et al. (2004) [233]	Food & nutrient	USA	1) 24 h recall	Reflects adherence to the 1998 Food Guide Pyramid for 2–6y for components relevant to public health [234]	8 components, scored 0–70, calculated by nutrient analysis & servings	2–5y	No
Revised Children’s Diet Quality Index (RC-DQI); Kranz et al. (2006) [235]	Food, nutrient & behaviour	USA	1) 24 h recall, 2) Food diary/record	Reflects adequacy of nutrients & food group intakes which are of a public health concern [140, 236, 237]	13 components, scored 0–95, calculated by nutrient analysis & servings	2–18y	No
Food Variety Index for Toddlers (VIT), Cox et al., 1997 [238]	Food & nutrient	USA	1) 24-h recalls 2) food diary/ records	Reflect dietary adequacy and adherence to food groups in the Food Pyramid [150] dietary guidelines recommendations and food groups.	5 components, ratio calculated from 0.00–1.00 for each, then a total VIT score was averaged.	24-36 m	No
Food Variety Index for children (VIC), Skinner et al., 1999 [239]						24-60 m	
The Healthy Eating Preference Index (HEPI); Sharafi et al. (2015) [240]	Food & nutrient	USA	1) Preschool-Adapted Liking Survey (PALS), and healthy variety score	Reflects adherence to Dietary Guidelines for Americans 2010 [185]	Scored − 250-250, foods categorised into liking groups and healthy variety score calculated, then conceptual weights were assigned to foods and adherence to dietary guidelines assessed.	Pre-school (3-5y)	Yes
Nutrient Rich Foods index (NRF) Drewnowski et al. (2009) [241]	Food & Nutrient	USA	1) 24 h recall	Reflects adherence to Dietary Guidelines for Americans and consumption of nutrient rich foods [242]	NRF^j^ 9.3 12 components, constructed from 9 encouraged nutrients minus 3 discouraged nutrients, calculated from percentage of reference intake	≥4y	No
Alternative Healthy Eating Index (AHEI); Chiuve et al. (2012) [243] developed in adults, Harris et al. (2016) [244] used in adolescents	Food & nutrient	USA	1) FFQ (HS-FFQ)	Reflects dietary patterns to lower risk of chronic disease [218] and US dietary Guidelines [222] based on validation of the index in previous studies [245–247]	9 components, scored 2.5–87.5, calculated by nutrient analysis & servings	13-18y	No
Youth Healthy Eating Index (YHEI); Feskanich et al. (2004) [248]	Food & behaviour	USA	1) FFQ	Reflects adherence to American Dietary Guidelines [249]	13 components, scored 0–100, points awarded & summed	Not specified	No
Alternative Healthy Eating Index (AHEI); McCullough et al. (2002) [245]	Food & nutrient	USA	1) FFQ	Reflects dietary patterns to lower risk of chronic disease [218] and US dietary Guidelines [219] Indicates diet diversity in adherence with development & analysis guidelines for developing countries [216, 217]	9 components, scored 2.5–87.5, calculated by nutrient analysis & servings	Not specified	No
Diet Quality Index (DQI); Patterson et al. (1994) [250]	Food & nutrient	USA	1) 24 h recall, 2) 24 h recall & food diary/ record	Reflects adherence to the 1989 Recommended Dietary Allowances [161]	8 components, scored 0–16, points awarded & summed	Not specified	No
Diet Quality Index-International (DQI-I); Kim et al. (2003) [251] developed in adults, Setayeshgar et al. (2017) [252] used in Canadian children	Food & nutrient	Designed in USA to be of international use	1) FFQ, 2) 24 h recall & FFQ	Reflects worldwide (WHO [209], USA [150, 210] & China [211, 212] adherence to dietary food & nutrient recommendations	4 major components with sub-components, scored 0–100, calculated by nutrient analysis & servings	Not specified	No
Grain, Fruit, Vegetables, Dairy & Mild (GFVDM) Variety Score; Falciglia et al. (2009) [253]	Food	USA	1) 24 h recall	Reflects adherence to the 1992 Food Guide Pyramid food groups [150]	5 food categories, servings summed	Not specified	No
Grain, Fruit & Vegetable (GFV) Variety Score; Falciglia et al. (2009) [253]	Food	USA	1) 24 h recall	Reflects the 2000 Dietary Guidelines for variety [249]	3 food categories, servings summed	Not specified	No
Modified KIDMED (M-KM); Wang et al. (2015) [67] abstract not described, Martin-Calvo et al. (2016) [254]	Food	USA	1) FFQ (YAQ)	Adherence to Mediterranean dietary pattern (MDP) [255]	16 components, scored 1, 0 or − 1, points awarded and summed for a total score of −4 to 12.	Not specified	No
Healthy Diet Score, Anderson et al. (2015) [256]	Food	USA	1) FFQ	Assesses healthy/unhealthy components of the diet independently. Binary variables based on US dietary recommendations for pre-schoolers [257].	Healthy diet score:3 components, scored 0–6, points awarded and summed. Unhealthy diet score: Scored 0–24 points awarded & summed.	Not specified	No
Adapted Complementary Feeding Utility Index (aCFUI); Au et al. (2018) [258]	Food & behaviour	USA	1) 24-h dietary recall	Reflects adherence to complementary feeding guidelines and the USDAinfant nutrition and feeding guidelines [201]	13 components, scored 0–13, by calculating the probability functions, then summed.	Not specified	No
A Priori Diet Quality Score (APDQS); Mursu et al. (2013) [259] developed in women, Hu et al. (2016) [260] used in adolescents	Food & nutrient	USA	1) FFQ (YAQ)	Reflects adherence to the Mediterranean dietary pattern (without alcoholic items) [255]	34 components, 13 beneficial, 12 adverse and 9 neutral, calculated as the sum of quintile scores 0–4 for beneficial foods plus scores in the reverse order (4–0) for adverse foods.	Not specified	No
Dietary Approaches to Stop Hypertension (DASH) diet score; Günther et al. (2009) [261]	Food & nutrient	USA	1) FFQ	Reflects adherence to DASH dietary pattern [145] and the dietary guidelines for Americans [140]	8 components, scored 0–80, calculated by reported intake/ recommendations, points awarded and summed	Not specified	No

*d* day, *DHQ* Dietary Habits Questionnaire, *FFQ* Food frequency questionnaire, *g* gram, *HS-FFQ* High school food frequency questionnaire, *h* hour, *INCAP* Institute of Nutrition of Central America & Panama, *m* month, *OMD* Optimised mixed diet, *NZ* New Zealand, *UK* United Kingdom, *USA* United States of America, *USAID* United States Agency for International Development, *USDA* United States Department of Agriculture, *WHO* World Health Organisation, *WIC* Women, Infants, and Children Nutritional Supplementation Program, *y* year, *YAQ* Youth and Adolescent Food Frequency Questionnaire

^a^Described as validated by the authors of the paper

^b^Toddler dietary questionnaire

^c^Finland, Italy, and Netherlands

^d^Belgium, Cyprus, Estonia, Germany, Hungary, Italy, Spain and Sweden

^e^Ireland and Italy

^f^Portugal, UK, France, and Greece

^g^Ghent, Belgium; Stockholm, Sweden; Vienna, Austria; Pecs, Hungary; Athens, Greece; Zaragoza, and Spain

^h^Vienna in Austria, Ghent in Belgium, Lille in France, Dortmund in Germany, Athens and Heraklion in Greece, Pe’cs in Hungary, Rome in Italy, Zaragoza in Spain and Stockholm in Sweden

^i^Spain, France, Germany Hungary, Greece, Italy, Belgium, Austria, Sweden, and United Kingdom

^j^Nutrient Rich Foods Index (NRF) can range from 5 to 15 nutrients

Most indices were scored by considering both food and nutrient intakes (*n* = 64 DQIs), while 34% (*n* = 44 DQIs) scored by considering food intake alone, and 6% (*n* = 7 DQIs [111, 114, 115, 117, 146, 148, 189]) scored using nutrient intake data alone (Table 2). In addition, 10% (*n* = 13 DQIs [46, 73, 75, 80, 121, 128, 165, 195, 214, 224, 235, 248, 258]) assessed a single behaviour (e.g. physical activity levels) as well as food and/or nutrient intake. The most common methods of collecting dietary data in studies which reported the development of DQIs were 24-h dietary recalls (*n* = 44) and food frequency questionnaires (FFQ) (*n* = 43); while some studies used both methods (*n* = 18), others used alternative methods such as study specific questionnaires or multiple day food diaries or records (*n* = 23) (Table 2).

A number of studies utilised information from the same datasets, such as data from the National Health and Nutrition Examination Survey (NHANES) prospective population surveillance in the USA, or the Healthy Lifestyle by Nutrition in Adolescence (HELENA) in Europe [263, 264].

### The quality and strength of papers identified

Of the 39 papers assessing validity and/or reliability of 37 DQIs, 22 papers had positive study quality, while 17 papers had neutral study quality (Table 3). Of the papers assessing the relationship with health-related outcomes, 10 papers had positive study quality and two papers had neutral study quality (Table 4). None of papers evaluated had a negative study quality. The most prevalent reasons for papers to be downgraded to neutral study quality was due to authors not reporting the eligibility criteria of participants, sampling method, or reasons for attrition.

**Table 3:** Studies evaluating the validity and/or reliability of paediatric a-priori diet quality indices (*n* = 37).

	Index	Study	Validation and reliability	Academy QCC rating
1	Australian child and adolescent recommended food score (ACARFS)	Marshall et al. (2012) [33] • Country: Australia • Age: 9-12y (μ 11.0, SD 1.1) • Sex: f 56.2% • Data collected: diet quality scores, food and nutrient intake and BMI • Data measurement: Cross-sectional	• Validity: Relative. • Reference standard: nutrient intakes and core food groups and demographics • Reliability: none. • Significant result (*p* < 0.001): ACARFS demonstrated statistically significant positive correlations with all vitamins and minerals tested. The strongest correlations were with vitamin C, β-carotene and fibre. ACARFS also had a moderately strong positive correlation with total energy. When the ACARFS was correlated with macronutrients adjusted for energy intake there was a positive correlation with protein. Weak negative correlation was found with total fat (*P* = 0.003) and SFA (*P* < 0.001). • The percent energy intake from SFA gave the least overall agreement of all the nutrients (κ = 0.13) and demonstrated ‘slight’ agreement, followed by riboflavin (κ = 0.36) which showed ‘fair’ agreement. Vitamin C (κ = 0.64), fibre (κ = 0.62) and β-carotene (κ = 0.62) had the strongest ‘substantial’ agreement. All other nutrients showed ‘moderate’ agreement (κ = 0.42–0.56). Within quartiles, fibre, vitamin C and β-carotene had the lowest percentages grossly misclassified. The strongest agreement amongst the quartiles was quartile one. • No association found between the ACARFS and percent energy intake from MUFA, PUFA, carbohydrate and sugar intake. • The percent energy intake from SFA gave the least overall agreement of all the nutrients (κ = 0.13) and demonstrated ‘slight’ agreement, followed by riboflavin (κ = 0.36) which showed ‘fair’ agreement. Vitamin C (κ = 0.64), fibre (κ = 0.62) and β-carotene (κ = 0.62) had the strongest ‘substantial’ agreement. All other nutrients showed ‘moderate’ agreement (κ = 0.42–0.56).	∅
2	Australian Recommended Food Scores for Pre-schoolers (ARFS-P)	Burrows et al. (2014) [19] • Country: Australia • Age: 2-5y • Sex: f 46% • Data collected: diet quality scores, food and nutrient intake • Data measurement: Cross-sectional	• Validity: Construct • Reference standard: nutrient intakes and core food groups, adjusted for total energy intakes and demographics • Reliability: none • Significant result (*p* < 0.05): positive association with protein, cholesterol, dietary fibre, vitamin A, beta-carotene, niacin equivalent, folate, vitamin C, Ca, Mg, K, P, Zn, vegetables, fruit, meat, and meat alternatives; and a negative association with carbohydrate, sugar sweetened drinks, packaged snacks, confectionary, take-away, and processed meats. • No association found with saturated fat, sugars, retinol, thiamine, riboflavin, Fe, Na, grains, dairy, baked sweet products, condiments, or sweet breakfast cereal.	+
3	Dietary Guideline Index for Children and Adolescents (DGI-CA)	Golley et al. (2015) [265] • Country: Australia • Age: 4-13y (grouped 4–8, 9–11, 12–13) • Sex: f 40% • Data collected: diet quality scores, food and nutrient intake and serum biomarkers • Data measurement: Validation study	• Validity: Concurrent/convergent • Reference standard: plasma dietary biomarkers and serum lipid concentrations via separate simple and multiple linear regression models, adjusted for demographic data • Reliability: none • Significant results (*p* < 0.05): Diet quality assessed by DGI-CA was a significant positive predictor of a-carotene, b-carotene, and n–3 FAs. Diet quality was inversely associated with lycopene and stearic acid (18:0) concentrations. • No association was found between diet quality and lutein and palmitic acid (16:0). DGI-CA had no association with lutein, a-tocopherol, n–6 FAs, myristic acid (14:0), pentadecanoic acid (15:0), palmitic acid, total cholesterol, cholesterol fractions, or triglycerides.	+
4	Obesity Protective Dietary Index (OPDI)	Spence et al. (2013) [43] • Country: Australia • Age: ~ 15 m • Sex: f 47% • Data collected: DQI score, nutrient intake and energy intake • Data measurement: Intervention, development and validation study	• Validity: Construct • Reference standard: Energy and nutrient intakes • Reliability: none • Significant results (*P* < 0.01): OPDI was positively correlated with intakes of energy (0.18), dietary fibre (0.55), b-carotene (0.51), and vitamin C (0.40). • No associations found between OPDI and intakes of saturated fat (20.02) or sodium (0.03). • When adjusted for energy intake, the correlations altered only for saturated fat (20.19) and sodium (20.11) and both were significant (*P* < 0.05).	∅
5	Short Food Frequency Questionnaire Diet Quality Index (sFFQ-DQI)	Kunaratnam et al. (2018) [45] • Country: Australia • Age: 2-5y • Sex: f 54.8% • Data collected: diet quality scores, anthropometry, food and nutrient intake and serum biomarkers of health and dietary exposure • Data measurement: cross-sectional validation study	• Validity: Comparative • Reference standard: sFFQ-DQI and the 3d-FR-DQI • Reliability: test-retest • Significant results (*p* < 0.05): There was a weak, but significant positive correlation between the sFFQ–DQI scores and 3d-FR–DQI scores. A positive mean difference occurred between sFFQ–DQI scores and 3d-FR–DQI scores and a significant positive trend indicating some bias between scores. Test-retest reliability of sFFQ–DQI scores and found no significant difference (*p* = 0.06) between mean total DQI scores. There was a high correlation between scores, Intraclass correlation (*p* < 0.001).	∅
6	Adapted Healthy Eating Index (adHEI)	Conceicao et al. (2018) [53] • Country: Brazil • Age: 1-2y • Sex: f 48.7% • Data collected: dietary scores and nutrient intake • Data measurement: Cross-sectional validation study	• Validity: Construct • Reference standards: adHEI components, diet *quality, dietary energy, demographics* • Reliability: internal consistency • Significant results (*p < 0.05)*: *The scores for adapted HEI components presented low correlations with energy intake, and correlation with individual food types was moderate, except in the case of milk and milk products. The correlations were negative for total fat, saturated fats, sodium, and cholesterol. The scores for the adapted HEI indicated a high positive correlation with dietary variety and vegetable consumption. For the other components of the index, the correlations ranged from moderate to low.*	∅
7	The Brazilian Healthy Eating Index-Revised (BHEI-R)	Toffano et al. (2018) [266] • Country: Brazil • Age: 9-13y • Sex: f 52.7% • Data collected: diet quality scores, food and nutrient intake and serum biomarkers of health and dietary exposure • Data measurement: Validation study	• Validity: Construct • Reference standard: BHEI-R dietary intake components, serum biomarkers and demographics • Reliability: none • Significant results (*p* < 0.04): Found between whole grains and 5 methyl tetrahydrofolate, vegetable and legumes intake were positively correlated with seven metabolites (LA, ALA, ARA, EPA, DHA, β-carotene and creatine). Dark green and orange vegetables (DGOV) and legumes were positively correlated with ALA, retinol, β-carotene, creatine DHA, retinol, β-carotene and S-adenosyl-homocysteine. Intake of total fruits positively correlated with LA, ALA, ARA, EPA, DHA and β-carotene. Whole fruits were only positively correlated with β-carotene and riboflavin. Milk and dairy were positively correlated with retinol and pyridoxal. Meat, eggs and legumes were positively correlated with ALA, DHA, and creatine. Negative significant correlations were found between saturated fat and retinol, and with α-tocopherol. • No significant associations (*p* ≥ 0.09): After adjusting results obtained for saturated fat with total cholesterol, no correlation was found for retinol or α-tocopherol.	+
8	Healthy nutrition score based on food intake for pre-schoolers (HNSP)^a^	Peng et al. (2015) [74] • Country: China • Age: pre-school children • Sex: Not specified • Data collected: food and nutrient intakes, serum biomarkers • Data measurement: Development and diagnostic study	• Validity: Construct • Reference standards: HNSP scores, nutrient intakes, serum nutrient levels and biochemical indicators • Reliability: none • Significant results (P ≤ 0.001): HNSP scores were positively associated with calcium, zinc, vitamin A, vitamin E, vitamin B1, vitamin B2 and vitamin C. • The Cronbach’s alpha score for the HNSP = 0.86, indicating good internal consistency. Inter-rater reliability and reproducibility, assessed via Cohen’s Kappa coefficient, scored 0.61, which indicates HNSP score had good reproducibility. • No significant results were seen between HNSP score and physical mass, BMI, age or age z-scores, blood biochemical indicators including haemoglobin or concentration of haemoglobin in red blood cells.	∅
9	Preschool dietary lifestyle index (PDL-index)	Manios et al. (2010) [121] Country: Greece • Age: 2-5y • Sex: f 48.5% • Data collected: BMI (OW & OB), food and nutrient intake • Data measurement: Development & validation study	• Validity: Construct • Reference standards: PDL index score and BMI score to validate associations between PDL-index score and BMI classifications. • Reliability: none • Significant results (*P* < 0.001): Consumption of vegetables, fruits, fish/seafood, unsaturated fats and white meats/legumes was significantly higher in participants belonging to the third tertile of the PDL-Index compared to those belonging to the lowest tertile. Red meat, sweets and grains was significantly lower in the third tertile compared to the first tertile. Total and saturated fat intake was significantly lower, while the protein and carbohydrate intake were significantly higher in the third compared to the first tertile. Participants who belonged to the third tertile of the PDL-Index were less likely to be OB or OW/OB compared to those who belonged to the first tertile.1/44 unit increase in score was associated with 5 and 3% lower odds of being OB and OW/OB, respectively. • No significant difference was detected in total energy intake across the tertiles of the index. No significant difference was detected in monounsaturated and polyunsaturated fat intake across the tertiles of the PDL-Index. The PDL-index was not strongly associated with fibre, zinc and riboflavin intake.	+
10	Healthy dietary-lifestyle index (HDL-index)	Manios et al. (2010) [130] • Country: Greece • Age: 10-12y • Sex: Not specified • Data collected: food and nutrient intake, medical examination including serum biomarkers of health (fasting glucose & fasting insulin) • Data measurement: Cross-sectional study	• Validity: Construct • Reference standard: diet quality, nutrient intake & insulin resistance and demographics • Reliability: none • Significant results (*p* < 0.001): Higher HLD-Index score was associated with lower proportion of children having intakes lower than EAR. Mean intake of fibre, calcium and vitamin K was significantly higher among schoolchildren in 3rd tertile of the index. Saturated fat intake was significantly lower among children with higher HLD-Index score (*p* = 0.029). 1/40 unit increase in the HLD-Index score was associated with almost 7% lower odds of being insulin resistant. The likelihood of being insulin resistant was almost 60% lower among participants with high HLD-Index score (3rd tertile) compared with those belonging to the 1st tertile. • No significant difference was detected in total, monosaturated and polysaturated fat, carbohydrate and protein intake across the tertiles of index.	∅
11	NutricheQ Tool	Rice et al. (2015) [87] • Country: Ireland • Age: 12-36 m • Sex: f 50% • Data collected: food and nutrient intake and anthropometric measurements • Data measurement: Validation study	• Validity: Concurrent • Reference standard: NutricheQ scores, nutrient density, anthropometrics and food group via analysis of covariance and demographics. • Reliability: test-retest • Significant results (p ≤ 0.05): Correlation analysis for section 1 revealed statistically significant, negative correlations between NutricheQ scores and seven nutrients (iron, vitamin D, zinc, thiamine, vitamin C, fibre, and saturated fat) and vegetables, the strongest correlation being for iron and vitamin D. Correlation analysis for section 2, statistically significant correlations were obtained for 14 nutrients (protein, fibre, SFA, non-milk sugars, Fe, Zn, Ca, riboflavin, folate, thiamine, P, K, carotene, and retinol) and for fruit and vegetables. When scores were combined (i.e. total score), similar statistically significant, weak correlations were maintained except for saturated fat and vitamin C. Analysis of energy-adjusted dietary intakes across the groups showed significant differences in mean daily intakes of most nutrients. Nutrient density was significantly lower for those with higher NutricheQ scores, ie. differences between the lowest and highest scoring groups were observed for dietary fibre, iron, vitamin D, and carotene patterns were supported by food group analysis where children in the highest scoring groups ate significantly less vegetables and vegetable dishes, fish/fish dishes and meat, and more non-milk beverages, processed foods and ‘sugars, confectionery, preserves and savoury snacks. • Levels of agreement for sensitivity SN and SP across a range of NutricheQ scores, ROC curves were generated based on high and moderate risk ratings, with an AUC for high risk of 85%, whereas the AUC for moderate risk was 76%. • Cronbach’s alpha subsequently returned a relatively low score of 0.5; however, it has been reported that values of 0.5 are satisfactory.	+
		Aramouny et al. (2018) [267] • Country: Lebanon • Age: μ: 22.2 m • Sex: f 45% • Data collected: DQI scores with age, gender, weight and BMI • Data measurement: Validation study	• Validity: Concurrent • Reference standard: NutricheQ questionnaire, average daily intake of nutrients • Reliability: none • Significant results (*P* < 0.05): Caffeine was positively associated with the NutrichQ score, the number of high-fat meats also was positively associated with the score. EPA was negatively associated with score, DHA was negatively associated with score, Fluoride and chromium were positively associated with the total score. Molybdenum was positively associated with risk score, soluble fibre was negatively associated with the score, lactose was positively associated with risk score. Lysine was negatively associated with risk score caffeine was positively associated with the score and fat was positively associated with total score.	∅
12	Healthy Eating Index for Malaysians (HEI-m)	Rezali et al. (2015) [175] • Country: Malaysia • Age: 13-16y • Sex: Not specified • Data collected: DQI score, food and nutrient intakes • Data measurement: Validation study	• Validity: Content • Reference standards: composite score of the HEI and adequacy of nutrient intakes • Reliability: none • Significant results (*P* < 0.05): The composite score of the HEI was significantly and positively correlated with adequacy of protein, calcium, thiamine, riboflavin, vitamin A, and vitamin C intakes, indicating that it can be used to assess diet quality.	∅
13	Diet quality score for preschool children	Voortman et al. (2016) [177] • Country: Netherlands • Age: 12-19 m • Sex: Not specified • Data collected: DQI score, food and nutrient intakes • Data measurement: Development and validation study	• Validity: Construct and predictive • Reference standards: five dietary patterns and each of the body composition measures, adjusted for energy intake and demographics • Reliability: none • Significant results (*p* < 0.05): ‘Health-conscious’ dietary pattern or a higher diet quality score at the age of 1 year was associated with a higher fat-free mass index at 6y -not associated with fat mass index or %BF. The first reduced-rank regression (RRR)-derived pattern, showed diet quality was positively correlated with FMI and FFMI, remained positively associated with both FMI and FFMI after adjustment for confounders and was also associated with a higher BF% and a higher android/gynoid ratio. The second RRR-pattern, showed diet quality was positively correlated with FFMI and inversely correlated with FMI, remained positively associated with FFMI (0.19 (95% CI 0.06; 0.32) SD for highest vs. lowest quartile) after adjustment, but was no longer significantly associated with FMI. • Non-significant results: Adherence to a ‘Western’ dietary pattern at the age of 1 year was not consistently associated with any of the body composition measures the age of 6y.	+
14	Diet quality score for school aged children	van der Velde et al. (2018) [178] • Country: Netherlands • Age: mean 8y • Sex: not specified • Data collected: diet quality score, food and nutrient intakes • Data measurement: Validation study	• Validity: Construct • Standard preferences: diet quality score, intake of nutrients and energy intake • Reliability: none • Significant results (*p* < 0.01): Positive correlation between the diet quality score and intakes of protein (mainly plant protein), dietary fibre, and n-3 fatty acids. The score was inversely correlated with intakes of saturated fat, and monosaccharides and disaccharides. The score was also positively correlated with intake of all of the examined micronutrients.	+
15	Dietary Index for a Child’s Eating (DICE)	Delshad et al. (2018) [268] • Country: New Zealand • Age: 2-8y • Sex: Not specified • Data collected: dietary scores and nutrient intakes • Data measurement: Validation study	• Validity: Relative and construct • Standard references: DICE and 4-day food record scores • Reliability: test-retest • Significant results (*p* < 0.05): A significant positive correlation was observed between the total scores for DICE and the 4DFR. The weighted ĸ-statistic demonstrated moderate agreement (ĸ = 0.49) between DICE and the 4DFR. Spearman’s correlation coefficients showed significant positive correlations between the DICE and 4DFR for servings of fruit, servings of vegetables, variety of vegetables, servings of bread and cereals, consumption of wholegrain products, servings of milk and milk products, servings of meat and its alternatives, number of meals and snacks, and fluid consumption. A significant and inverse correlation was found for low fat foods/snacks/drinks consumption. Higher intake of fibre, vitamin C, vitamin A, vitamin D, folate (*p* < 0.05), and calcium (*p* < 0.001) were associated with increasing tertiles of the DICE total score. There was no bias between the two methods; that is the difference in intake between the DICE and 4DFR did not alter across the mean intake • Non-significant results: The variety of fruits, low salt and low sugar foods/snacks/drinks components were not significantly correlated with the same components scores from the 4DFR.	∅
16	Complementary Feeding Utility Index (CFUI)	Golley et al. (2012) [80] • Country: UK • Age: 3y • Sex: Not specified • Data collected: diet quality score and nutrient intakes • Data measurement: Validation study	• Validity: Concurrent/convergent • Reference standards: CFUI, dietary intake, feeding behaviour • Reliability: none • Significant results (*p* < 0.01): Higher CFUI scores were associated with higher energy-adjusted intakes of polyunsaturated fat, carbohydrate, total sugar (including fruit sugar), fibre, non-starch polysaccharide, and folate. • Higher index scores were also associated with lower energy-adjusted intakes of protein, calcium, and iodine.	+
		Golley et al. (2013) [269] • Country: UK • Age: 7-8y • Sex: f 48.3% • Data collected: food and nutrient intakes, anthropometry, BP, lipids • Data measurement: observational prospective cohort and validation study	• Validity: Predictive • Reference Standards: CFUI score, dietary patterns, BP, blood cholesterol and demographics. • Reliability: none • Significant results (*p* < 0.001): Greater adherence to complementary feeding guidelines (i.e., higher CFUI score) was negatively associated with the processed dietary pattern and positively associated with the health-conscious dietary pattern at 7y. • In the unadjusted models, CFUI score was negatively associated with BMI and waist circumference however, in fully adjusted model, the point estimates for both associations were attenuated by about one-half and only a weak association with waist circumference remained (*p* = 0.046). Results were consistent when stratified by gender. Similar inverse associations were observed between CFUI score and both systolic and diastolic BP (*p* < 0.05). • Non-significant associations: CFUI score was not associated with total cholesterol or cholesterol fractions in either the unadjusted or fully adjusted models or the gender-stratified analyses. CFUI score was not associated with the traditional dietary pattern. Stratified for gender, CFUI was weakly associated with the traditional dietary pattern in boys (*p* = 0.008) but not girls (*p* = 0.91).	+
17	Healthy Eating Index-2005 (HEI-2005)	Kranz et al. (2013) [152] • Country: USA • Age: 2-18y • Sex: Not specified • Data collected: DQI scores, food and nutrient intakes • Data measurement: Validation study	• Validity: Content and construct • Reference standards: HEI-2005, nutrient intakes • Reliability: none • Significant results (*p* < 0.05): associations were seen between: dairy and whole grains, dairy and fruit, whole grains and total grains, whole grains and fruit, total grains and vegetables, and total grains and fruit. The RC-DQI Analysis of the correlation between component scores in the RC-DQI showed that all comparisons were positive. The correlations between the identical components, that is, RC-DQI dairy and HEI 2005 dairy, were positive. • Non-significant results: association between dairy and total grains, vegetables or whole grains and vegetables, and vegetables and fruit.	+
18	Healthy Eating Index (HEI) & Youth Healthy Eating Index (YHEI)	Hurley et al. (2009) [270] • Country: USA (African American Adolescents) • Age: 11-16y • Sex: f 49% • Data collected: DQI score, nutrient intake, BMI, %BF • Data measurement: Validation study	• Validity: Concurrent • Reference standards: HEI, YHEI and health indicators • Reliability: none • Significant results (*p* < 0.05): Both HEI and YHEI, had significant positive correlations between index scores, micronutrients and total energy intake. In the Challenge sample, the magnitude of the correlation was significantly higher for the HEI vs. YHEI for iron. Among Challenge participants, higher percent body fat and abdominal fat were associated with a lower overall HEI score. • Non-significant results: BMI and total HEI or YHEI scores were not significantly associated. However, the directions of the associations were consistent with our hypothesis.	+
19	Diet Quality Index for Preschool Children (DQI-CH)	Huybrechts et al. (2010) [46] • Country: Belgium • Age: 2.5–6.5y • Sex: Not specified • Data collected: DQI scores, nutrient intakes • Data measurement: Validation and reproducibility study	• Validity: Construct and relative • Reference standards: DQI scores, nutrient intakes and 3d estimated diet records • Reliability: test-retest • Significant results (*p* < 0.05): The dietary diversity score was positively associated with vitamin C, thiamine, riboflavin, Na, K, Ca, P, Mg and Zn intakes, total water, fibre, protein and SFA intakes. The dietary quality score is negatively associated with energy, MUFA and carbohydrate intakes, while it was positively associated with thiamine, riboflavin, K, Ca, P and Mg intake, and protein, water and fibre intakes. Dietary equilibrium score was inversely correlated with energy, total fat, carbohydrate, MUFA and PUFA intakes, while it was positively correlated with protein, fibre, water, riboflavin, Ca, P, Mg and Zn intakes. The meal index was positively associated with energy, PUFA, complex carbohydrates, fibre, Na, Fe and Mg intakes. • No significant differences in mean DQI scores for preschool children were found between repeated measurements in the reproducibility study • The validity correlation for the DQI score corrected for within-individual variability was 0·82. Pearsons correlations varied among the four main components of the DQI (from 0·39 to 0·74)	∅
20	Modified revised children’s diet quality index (M-RCDQI)	Keshani et al. (2018) [151] • Country: Iran • Age: 13-15y • Sex: f 46.7% • Data collected: DQI score & nutrient intakes • Data measurement: cross sectional	• Validity: Content • Reference standards: M-RCDQI diet quality components • Reliability: test-retest • Significant results (*p* < 0.03): • Adolescents’ diet quality had positive significant association with HBM constructs, cues to action and self-efficacy. For every unit increase in cues to action score, a 0.19 unit increase in M-RCDQI was predicted, holding all other variables constant. Evaluating the relationships between cues to action and M-RCDQI components, we found a positive, significant association between cues to action and fruit consumption. A negative significant association was observed between cues to action and total fat intake and linoleic acid. For every unit increase in cue to action score, a 0.62 unit decrease in fat intake was predicted, holding all other variables constant. Furthermore, self-efficacy had a direct significant association with dairy intake	+
21	Finnish Children Healthy Eating Index (FCHEI)	Kyttälä et al. (2014) [104] • Country: Finnland • Age: 1, 3 & 6y • Sex: Not specified • Data collected: energy intake, energy adjusted intakes of SFA, MUFA, PUFA, dietary fibre and sugars, as well as absolute intakes of vitamin D and E • Data measurement: development and validation study	• Validity: Relative • Reference standards: diet quality score and nutrient intakes • Reliability: none • Significant results (*p* < 0.04): High amounts of sugar’ correlated positively with the scores of ‘vegetables, fruits and berries’, ‘oils and margarine’ and ‘skimmed milk’ at 1y, 3y and 6y. The score of ‘fish and fish dishes’ correlated positively with the scores of ‘vegetables, fruits and berries’ among the 3y and ‘oils and margarine’ among 6y. In all ages, energy adjusted intakes of SFA and sugars decreased across ascending quartiles of the FCHEI scores. Further, the energy density of the diet was lower among those 3y and 6y who belonged to the higher FCHEI quartiles. Strong inverse correlations of SFA, sugars and energy density of the diet with the FCHEI scores indicate that a higher FCHEI reflects a healthier diet. Energy-adjusted intakes of PUFA and dietary fibre, as well as absolute intakes of vitamins D and E, increased across ascending quartiles of the FCHEI scores in all age groups. Energy-adjusted intakes of PUFA and dietary fibre had strong positive correlations with the FCHEI scores. Absolute intakes of vitamin D and vitamin E correlated positively with the FCHEI.	+
22	Electronic Kids Dietary Index (E-KINDEX)	Lazarou et al. (2011) [271] • Country: Cyprus • Age: 9-13y • Sex: f 58.64% • Data collected: DQI scores, nutrient intake and body composition • Data measurement: Development study	• Validity: Predictive • Reference standards: E-KINDEX score, BMI classification and waist circumference • Reliability: none • Significant results (*p* < 0.001): Each 1 SD increase in the E-KINDEX score was associated with a 2.31 ± 0.23 kg/m2 decrease in BMI, a 2.23 ± 0.35 decrease in calculated %BF, and a 2.16 ± 0.61 cm decrease in WC. Significant and consistent inverse associations between the E-KINDEX score and BMI, %BF, WC, and generalized Obesity were observed in all models. • Overall, the diagnostic ability of the score appears more effective in screening for OB than for OW status in this sample. Compared with children belonging to the lowest E-KINDEX category those with scores in the second, third, and fourth categories had, on average, a 73, 76, and 85% decreased likelihood of being OW/OB, respectively. Children with scores that fell into the second, third, and fourth categories were, respectively, 62, 78, and 86% less likely to exhibit WC ≥ 75th percentile Being classified in the highest scored category was associated with an 84% decreased likelihood of an increase in BMI greater than 3 kg/m2 in 1 year (OR, 0.16; 95% CI, 0.04–0.74).	∅
23	Food Index (FI)	Magriplis et al. (2015) [122] • Country: Greece • Age: 10-12y • Sex: Not specified • Data collected: DQI scores, weight, BMI, %BF • Data measurement: Cross-sectional study	• Validity: Construct • Reference standards: FI score, percentage of body fat %, fat mass, BMI • Reliability: none • Significant results (*p* < 0.05): A difference was found in the gender’s mean BMI, WC and in total Energy intake. Difference was found assessing BMI categories between boys and girls, ~ 57% of boys versus 60% of girls being under- or normal-weight; 30% boys versus 29% girls were OW; and 13% boys versus 11% girls were OB. A borderline difference between BMI categories and age groups was found. • Associations were found between total food score and BMI, and their WC, in a crude analysis. When stratified by gender, the association remained significant for both genders for BMI (boys: −0.058 ± 0.03, 95% CI: −0.012, −0.001; girls: −0.06 ± 0.04, 95% CI: −0.016, −0.004) but only for girls in the case of WC (boys: −0.075 ± 0.04, 95% CI: −0.158, 0.008; girls: −0.098 ± 0.01, 95% CI: −0.177, −0.019). With every unit increase in the FI score the children were − 0.057 times less likely to be OW or OB and 0.08 less likely to have a high WC. The strength of the association remained significant in both the cases, when adjusted for confounders. BMI category increases the total FI score is lower than the median FI score. Gender, age and inactivity provided significant results. Sensitivity analysis that tested the probability of children being OW/OB with the total FI score showed that as the FI total increases in the 25% randomly selected GRECO sample, the probability of OW/OB decreases significantly. • Non-significant results: Total energy intake is entered BMI categories have no significant association with the dichotomized FI score.	+
24	Infant and Child Feeding Index (ICFI).	Moursi et al. (2009) [272] • Country: Madagascar • Age: 6-23 m • Sex: Not specified • Data collected: DQI scores, nutrient intake, energy intake, Length-for-age score, • Data measurement: validation study	• Validity: Concurrent and construct • ICFI scores, mean micronutrient density adequacy and energy intake • Reliability: none • Significant results (*p* < 0.0001): Complementary food energy intake increased with age. MMDA also increased with age. Both energy intake from complementary food and mean micronutrient density adequacy were positively correlated with ICFI across all age groups. Contrastingly, mean ICFI decreased with age and was the lowest for children between 12 m and 24 m of age. Both energy intake from complementary food and MMDA were positively correlated with ICFI across all age groups. Breastfeeding was overall significantly associated with LAZ with a .0.16 z-score difference in favour of non-breast-fed children. Dietary diversity was associated with LAZ when all age groups were combined with higher dietary diversity translating into better mean LAZ. There was a strong difference of 0.45 z-score when moving from medium to high frequency of feeding in 9–11 m children (P.0.01), but differences became marginally significant when all age groups were combined. • Non-significant results: There was no association between either WAZ or WLZ and ICFI after adjustment for specific confounders. Although statistically significant associations occurred between the ICFI and LAZ in the univariate analysis (P.0.002), it did not remain significant after adjustment • The exception to that was the 6–8 m age group for which there was a .0.65 LAZ difference for children with high ICFI compared to those with low ICFI (P.0.02).	+
25	Healthy Eating Index (HEI) for Brazilians	Rauber et al. (2014) [50] • Country: Brazil • Age: 3-4y & 7-8y • Sex: f 43% • Data collected: DQI scores, serum biomarkers • Data measurement: Development and validity study	• Validity: Construct • Reference standards: HEI scores and HEI components, energy, and nutrients • Reliability: none • Significant results (*p* < 0.05): At 3-4y, the food groups and dietary variety increased across the HEI score quartiles (from the lowest to the highest), except for the milk group, whereas intake of total fat, saturated fat, and sodium decreased. At 7-8y, food groups and dietary variety increased across the HEI score quartiles, whereas total fat, saturated fat, and sodium intake decreased. Contrary to expectations, cholesterol intake was positively correlated to the HEI score. The selected nutrients correlated to the HEI score, except for vitamin B12 at 3-4y, energy and carbohydrates at 7-8y, and calcium in both age groups.	∅
26	Diet Quality Index Score (DQIS)	Rios et al. (2016) [200] • Country: Puerto Rico • Age: 0-24 m • Sex: f 46% • Data collected: DQI score and BMI • Data measurement: Cross-sectional study	• Validity: Relative • DQIS categories and weight status and demographics • Reliability: none • Significant results it was found a trend, between DQIS categories and weight status, in which those categorized as having ‘Poor’ diets had two-fold higher odds of Excessive weight compared to those categorized as having ‘Excellent’ diets, after controlling for caregiver’s age and education (OR 2.01; 95% CI: 0.85, 5.18).	+
27	Menzies remote short-item dietary assessment tool (MRSDAT)	Rohit et al. (2018) [30] • Country: Australia (remote aboriginal communities) • Age: 18-54 m • Sex: Not specified • Data collected: ability and ease of completing index & DQI scores • Data measurement: development and validation study	• Validity: None • Reference standards: Diet scores and nutrient intakes • Reliability: Test-retest • Significant results: Test–retest analysis showed good-to-very good agreement between participant responses for 20 of the 24 items tested (0.63–0.88). The four items that showed weak agreement (0.13–0.50) were for questions regarding homemade freshly squeezed juice, red meat serve size, offal consumption and the frequency of consuming confectionery (chips, chocolates and ice creams). The MRSDAT was then modified to address these issues.	∅
28	Menzies remote short-item dietary assessment tool (MRSDAT)	Tonkin et al. (2018) [28] • Country: Australia (remote aboriginal communities) • Age: 6-24 m • Sex: f 50% • Data collected: DQI score from MRSDAT • Data measurement: Validation study	• Validity: Relative • Reference standards: MRSDAT scores, 24-h recalls • Reliability: none • Significant results: Relative to the 24-h recalls, the MRSDAT had higher estimates across all food groups, except fruit. While the median reported intakes for vegetables differed by only 0.04 servings between the two methods, and breads and cereals differed by 1.19 servings per day, Wilcoxon signed-rank test only showed the meat and vegetable intakes to be significantly different (*p* < 0.001 and *p* = 0.04, respectively). • Significant results (*p* < 0.05): Small bias reflects that the MRSDAT-estimated DGI-CA scores were both higher and lower to a similar degree compared with those derived from 24-h recalls. Secondary analyses showed that the MRSDAT-estimated DGI-CA scores were higher compared with 24-h recalls for all participants. Secondary analyses of individual dietary indicators showed significantly higher scores for meat and wholegrain indicators, and significantly lower dietary variety scores, when estimated by the MRSDAT compared with scores derived from 24-h recalls. For the meat indicator score, this bias was proportional; with the increasing indicator score, the difference between the MRSDAT and 24-h recalls scores was reduced. Regression for the wholegrain indicator showed a borderline-significant proportional bias in the opposite direction and this was also the case for the breads and cereals indicator; with increasing indicator scores, the difference between MRSDAT and 24-h recall derived scores increased. Given discretionary indicator is negatively scored, lower MRSDAT discretionary indicator scores are consistent with the MRSDAT, tending to estimate higher intakes of all foods. Kappa showed there was moderate agreement between methods for determining whether a child is still breastfed.	∅
29	Children’s Index of Diet Quality (CIDQ).	Röytiö et al. (2015) [100] • Country: Finland • Age: 2–6 • Sex: f 52% • Data collected: DQI scores • Data measurement: development and validation study	• Validity: Concurrent • Reference standards: CIDQ cut off scores and nutrient intake values • Reliability: none • Higher CIDQ scores were related to higher proportions of energy from protein (*P* = 0.001) and carbohydrates (*P* = 0.005) and lower proportions of energy from fat (*P* = 0.001), SFA (*P* = 0.001) and saccharose (*P* = 0.007). Higher intake of fibre (*P* = 0.001) and decreased intake of cholesterol (*P* = 0.001) were also associated with greater index scores and thus a good-quality diet. Of the several calculated intakes of different vitamins and minerals, higher intakes of Fe (*P* = 0.02), vitamin C (*P* = 0.001), vitamin E (*P* = 0.02) and folate (*P* = 0.001) were related with higher CIDQ points. Intakes of Ca and vitamins C and E increased from the lowest index group to the moderate and further to the highest group, which reflected healthier diet quality. The intake of SFA (E%) decreased when moving from the lowest group to the moderate and highest groups. Intake of MUFA did not change according to the three diet quality categories. • Analysis of the biochemical markers demonstrated that higher CIDQ scores were associated with clinical biomarkers that are connected with health, such as cholesterol (*P* = 0.008) and vitamin C (*P* = 0.008) concentrations. The children in the highest CIDQ group, which described good diet quality, had the lowest serum total cholesterol (*P* = 0.008) and LDL cholesterol (*P* = 0.02) concentrations and these concentrations increased significantly when moving down to moderate and low diet quality index scores. However, the same was detected also for HDL cholesterol (*P* = 0.01) concentrations. Vitamin C concentration increased significantly from the lowest to the highest diet quality category (*p* = 0·008). • Children’s BMI was not was not associated with the CIDQ score (r = 0·03, *P* = 0·65). The proportion of children with overweight (BMI ≥ 25·0 kg/m2) was 22·8%, 20·3% and 20·0% in the CIDQ score categories of poor (< 10 points), moderate (10·0–13·9 points) and good (≥14 points) diet quality, respectively (*P* = 0·86). No association was observed between the number of fulfilled criteria of healthy diet and overweight. The proportions of children with overweight was 24·1%, 20·4% and 18·9% when zero to two, three or four, or five or six criteria were fulfilled, respectively (*P* = 0·70).	+
30	The Healthy Eating Preference Index (HEPI)	Sharafi et al. (2015) [240] • Country: USA • Age: 2-5y • Sex: f 48% • Data collected: DQI scores, nutrient intake, energy intake and BMI • Data measurement: Validation	• Validity: Construct, predictive and concurrent • Reference standards: components of HEPI and energy intake and demographics • Reliability: Internal consistency • Significant results (*p* < 0.05): All HEI components showed weak-to-strong significant associations with energy intake, except nonsignificant associations for whole and refined grains. PCA analysis of HEPI components showed multiple dimensions with adequate internal consistency (a = 0.74). HEI only approached adequate internal consistency (a = 0.45). Liking/intake discordance for high-fat/sweet/salty foods also predicted BMI percentiles highest percentiles were observed in the high/low group, whereas the lowest percentiles were in the low/low group. ANCOVA showed significant effects of ratio group on BMI percentiles. Pre-schoolers in the highest ratio grouping had the lowest BMI percentiles. Ratio groupings also predicted carotenoid status pre-schoolers liking a healthy diet equal or above the pleasurable activities had the highest carotenoid status versus those liking a healthy diet half as much as the pleasurable activities. Similarly, the ratio groupings were formed for liking of high-fat/sweet/salty foods to pleasurable activities (each group included at least 20% of pre-schoolers), with a significant main effect on BMI percentiles. When HEPI and HEI were combined into a latent dietary quality variable, the best model fit with stronger associations was observed. Hierarchical regression analysis showed that only the HEPI significantly explained BMI percentile as an alternative or added-value predictor. Although the HEPI and HEI were significantly correlated, discord was observed in 40% of pre-schoolers. A similar pattern of association and discord was noted for high-fat/sweet/salty foods. • Non-significant results: HEPI components showed associations with energy intake (Pearson’s rs, < 0.12). HEI did not significantly predict BMI percentiles.	∅
31	Probability of adequate nutrient intake (PANDiet) score	Verger et al. (2016) [107] • Country: UK • Age: 12-18 m • Sex: f 49.4% • Data collected: DQI scores, food intake, nutrient intake and energy intake • Data measurement: validation study	• Validity: Content and construct • Reference standards: PANDiet score and its components • Reliability: none • Significant results (*p* < 0.05): The mean probabilities for avoiding excessive Na and SFA intakes were very low: 0·13 (SE 0·01) and 0·12 (SE 0·01), respectively. The Spearman correlation between the PANDiet score and energy intake was very weak. The lower the PANDiet score, the higher the intakes of whole milk, sugar, preserves and confectionery, burgers, kebabs, sausages, meat pies and pastries, biscuits and soft drinks and the lower the intakes of vegetables, fruits, and formula. PANDiet scores were significantly different across the four groups but energy intakes did not differ. Compared with other groups, the children in the YCF+/CIF− and YCF+/CIF+ groups had better nutrient adequacy for SFA, PUFA, vitamin D, Zn, Fe and Cu. The intakes of vegetables, fruit, fish and water were not significantly different between the four groups.	+
32	Diet quality index for adolescents (DQI-A)	Vyncke et al. (2013) [88] • Country: ten European cities ^b^ • Age: 12.5–17.5y • Sex: f 52.3% • Data collected: DQI scores, food and nutrient intake, serum biomarkers • Data measurement: Validation study	• Validity: Construct • Reference standards: DQI-A score, food and nutrient intakes, serum biomarkers and nutritional status • Reliability: none • Significant results (*p* > 0·0005): A strong positive association between the DQI-A score and water intake was observed. Soft drinks, fruit juices and alcoholic beverages had significant negative associations with the DQI-A. DQI-A score and bread/cereals had a positive association. Milk and cheese were positively associated with the DQI-A score, and animal fat and vegetable fat showed a small, however, significant positive association with DQI-A. No significant relation was present with meat, fish, eggs and substitutes. All non-recommended (energy-dense and low-nutritious) foods showed a significant negative association with the DQI-A score. A positive association was observed between the DQI-A and water and fibre intake, and a negative relationship was found with total energy intake. Polysaccharides were positively related to the dietary quality, whilst intake of mono- and disaccharides showed a negative relationship. Minerals Na, K, Cl, Ca, Mg, Zn, F, I, P, Mn were positively associated with the DQI-A score. Intake of vitamins, thiamine, riboflavin, pantothenic acid, pyridoxine, biotin, folic acid, cobalamin, retinol equivalents, vitamin D and vitamin K showed a significant positive association with the calculated index. • Non-significant results: but no significant association between DQI-A and potatoes and grains. No significant association was seen between DQI-A and protein intake or fat intake. Fe and Cu were not associated with the DQI-A score. Vitamins niacin, vitamin C and vitamin E, did not show a significant positive association with the calculated index.	+
33	Diet Quality Index for NZ adolescents (NZDQI-A)	Wong et al. (2013) [192] • Country: New Zealand • Age: 14-18y • Sex: f 61% • Data collected: DQI scores, nutrient intakes • Data measurement: Development and validation	• Validity: Construct and relative • Reference standards: NZDQI-A, nutrient intakes and 4DFR • Reliability: test-retest • Significant results (*p* < 0.05): Comparing nutrient intakes across the thirds of NZDQI-A score, those in the top third had higher intakes of iron and lower intakes of total fat, SFA and MUFA. Higher total scores were also associated with higher total sugars and fructose in the trend analysis. NZDQI-A had a fair internal consistency in measuring diet quality. The NZDQI-A total score derived from the repeated FQs showed good reproducibility, with reliability coefficients ranging from 0.32 to 0.67 for the individual components. Test-retest reliability was highest for fruit, but lowest for the meat component.	∅
34	Healthy Dietary Habits Score for Adolescents (HDHS-A) ratios	Wong et al. (2014) [194] • Country: New Zealand • Age: 15-18y • Sex: f 53% • Data collected: DQI scores, nutrient intakes, nutrient outputs, anthropometric data and serum biomarkers • Data measurement: development and validation	• Validity: Construct • Reference standards: HDHS-A scores, 24-h nutrient intakes, nutritional biomarkers and demographics. • Reliability: Internal • Significant results (*p* < 0.05): HDHS-A score was negatively associated with energy intake; all nutrients were adjusted for total energy intake. Higher relative intakes of protein, dietary fibre, PUFA, and lactose and lower intakes of sucrose were associated with increasing thirds of HDHS-A. Associations in the expected directions were also found with most micronutrient intakes, urinary sodium excretion, and whole-blood, serum, and RBC folate concentrations. The items in the HDHS-A had low intercorrelations. Correlations between individual items with the total score were highest for intake of potato and root vegetable fries, followed by item soft drink/energy drink consumption. Overall indicating the HDHS-A index had good internal reliability.	∅
35	Norwegian Adolescent Diet Score	Handeland et al. (2016) [195] • Country: Norway • Age: 14-15y • Sex: f 52.5% • Data collected: DQI score • Data measurement: Development and reliability	• Validity: None • Reference standards: diet score and components • Reliability: test-retest • Significant results (*p* < 0.001): The real percentage agreement for the Diet Score (87.6%) and the indicators (74.0–91.6%) exceeded expected agreement for all parameters, and Cohen’s k was > 0.4 for all parameters, except red meat (k = 0.249).	+
36	Unnamed Diet quality index for muti-ethnic Asian toddlers	Chen et al. (2019) [172] • Country: Singapore • Age: 18 m • Sex: f 48.5% • Data collected: DQI score, food and nutrient intakes, energy intakes • Data measurement: Development and validation study	• Validity: Construct • Reference standards: DQI scores, National recommended food group scores, food intake and demographics • Reliability: none • Significant results (*p* < 0.001): Those in the high DQI tertile were more likely to meet the recommended servings of the basic food groups, as compared with those in the low score tertile; significant for all basic food groups, except total milk and dairy products (*p* = 0.26). Increasing trends of participants meeting recommendation for whole grains intake and moderation of foods high in sugar across tertiles. Those in the high score tertile tended to meet the RDA of dietary fibre, protein, calcium and vitamin A, compared to the low tertile, but no significant association was observed for the AMDR of macronutrients (carbohydrates, total fat and saturated fat) and RDA of iron. When nutrients were modelled as continuous variables, we observed that toddlers in the high score tertile had a lower proportion of energy intake from carbohydrates and a higher proportion of energy intake from protein. When DQI was modelled as a continuous variable for the abovementioned analyses, similar associations were observed. Both FFQ and 24-h recall data, we observed higher DQI-24 h score across tertiles of DQI-FFQ score. Macronutrient intakes estimated from 24-h recall, toddlers in the high DQI-FFQ score tertile had a lower proportion of energy intake from carbohydrates and a higher proportion of energy intake from protein • Non-significant results: No significant associations observed for dietary fats (total, saturated, monounsaturated and polyunsaturated fat), iron and calcium. High DQI tertile did not meet the recommended servings for total milk and dairy products, as compared with those in the low score tertile; significant for all basic food groups, except (*p* = 0.26). There was no significant association observed for toddlers in the high DQI-FFQ score tertile and the proportion of energy from total dietary fats.	+
37	The Chinese Children Dietary Index (CCDI)	Cheng et al. (2016) [73] • Country: China • Age: 7-15y • Sex: f 49% • Data collected: DQI score, energy intake, food and nutrient intake, anthropometry, physical activity levels • Data measurement: Development and validation study	• Validity: Relative • Reference standards: CCDI score, BMI, inactivity and dietary intake and demographics: • Reliability: none • Significant results (*p* < 0.05): Positive correlations of the CCDI with majority of nutrient adequacy ratios and mean adequacy ratios was demonstrated. Whole grain intake and frequency of fried foods were not significantly associated with the CCDI.	+

*ALA* α-linolenic fatty acid, *AMDR* Acceptable macronutrient distribution range, *ANCOVA* Analysis of covariant, *ARA* Arachidonic fatty acid, *BP* Blood pressure, *BMI* Body mass index, *Ca* Calcium, *CCDI* The Chinese Children Dietary Index, *CFUI* Complementary Feeding Utility Index, *CI* Confidence interval, *CIDQ* Children’s Index of Diet Quality, *CIF* Child infant formula, *Cl* Chloride, *Cu* Copper, *DHA* Docosahexaenoic acid, *DQI* Diet quality index, *DQI-A* Diet quality index for adolescents, *EPA* Eicosapentaenoic acid, *F* Fluoride, *f* female, *Fe* iron, *FFQ* Food frequency questionnaire, *FFMI* Fat free mass index, *FMI* Fat mass index, *FQ* Food questionnaire, *h* hours, *HBM* health belief model, *I* Iodine, *K* Potassium, *LA* Linoleic fatty acid, *LAZ* Length/Height-for-age Z Score, *m* months, *Mg* Magnesium, *MMDA* Mean micronutrient density adequacy, *Mn* Manganese, *MUFA* Monounsaturated fatty acids, *Na* Sodium, *OB* Obese, *OR* Odds ratio, *OW* Overweight, *P* Phosphorus, *PCA* Principle Component Analysis, *PUFA* Poly unsaturated fatty acids, *QCC* Quality criteria checklist, *RBC* Red blood cells, *RC-DQI* Revised Children’s Diet Quality Index, *RDA* Recommended dietary allowances, *RRR* Reduced-rank regression, *SE* Standard error, *SFA* Saturated fatty acid, *SN* Sensitivity, *SP* Specificity, *WAZ* Weight for Age Z Score, *WC* Waist circumference, *WLZ* Weight-for-length Z Score, *y* years, *YCF* Young child formula, *Zn* Zinc, *3d-FR-DQI* 3-day food records diet quality index, *4DFR* 4 day food records, *%BF* % body fat.

^a^Paper published in Mandarin, unable to translate via google, translated and results reported by a colleague.

^b^Vienna in Austria, Ghent in Belgium, Lille in France, Dortmund in Germany, Athens and Heraklion in Greece, Pe’cs in Hungary, Rome in Italy, Zaragoza in Spain and Stockholm in Sweden.

**Table 4:** Association of diet quality indices with prospective health-related outcomes in paediatric populations (*n* = 12).

Index	Study	Setting	Study quality	Health-related results
Diet Quality Index for Adolescents (DQI-A)	Vyncke et al. (2013) [88]	- Austria, Belgium, France, Germany, Greece, Hungary, Italy, Spain, Sweden. - μ14.7 (SD: 1.2) y; 52.6%F - Data set: ‘Healthy Lifestyle in Europe by Nutrition in Adolescence (HELENA) (2006–2007)	+	**Anthropometry:** No significant association. **Nutritional biomarkers:** Adjusted models: DQI-A scores positively associated with plasma 25(OH)D nmol/l (β = 0.005, 95% CI = 0·002, 0·008, *p* < 0.0001) and holo-transcobalamin pmol/l (indicator of B12) (β = 1·005, 95% CI = 1·002, 1·007, *p* = 0.0002) and n-3 FA status μmol/l (β = 0·376, 95% CI = 0·105, 0·646, *P* < 0·007).
Dietary Approaches to Stop Hypertension (DASH) diet score	Barnes et al. (2013) [273] [274]	- USA - T1DM: μ14.7 (SD: 3.1) y; 46.8%F - T2DM: μ16.8 (SD: 2.8)y; 65.4%F - Data set: SEARCH for Diabetes in Youth Study	+	**Anthropometry:** No significant association. **Blood pressure:** DASH score negatively associated with SBP (β = − 2.02, SE = 0.97, *p* = 0.0406) in T2DM sample. **HbA1c:** DASH score negatively associated with HbA1c% (β = − 0.2, SE = 0.07, *p* = 0.0063) in T1DM sample. **Lipid profile:** No significant association.
Healthy nutrition score based on food intake for pre-schoolers (HNSP)	Peng et al. (2015) [74]	- China - μ4.5 (SD: 0.87) y; 54%F - Data set: N/A	∅	**Anthropometry:** No significant association. **Nutritional biomarkers:** HNSP scores significantly associated with plasma retinol (r = 0.128, *p* = 0.004). No other significant associations.
Complementary Feeding Utility Index (CFUI)	Golley et al. (2013) [269]	- UK - Baseline: 6 m (48%F), follow-up: 7 (49%F) & 8y (50%F) - Data set: Birth cohort from the Avon Longitudinal Study of Parents and Children (ALSPAC)	∅	**Anthropometry:** Simple model: CFUI score was negatively associated with BMI (β = −0.13 [− 0.20, − 0.07], *p* < 0.001) and WC (β = − 0.31 [− 0.45, − 0.17], *p* < 0.001). Adjusted models: CFUI score was negatively associated with WC (β = − 0.15 [− 0.31, − 0.002], *p* = 0.046). **Blood pressure:** Simple models: CFUI score was negatively associated with SBP (β = − 0.66 [− 0.95, − 0.38], *p* < 0.001) and DBP (β = − 0.42 [− 0.63, − 0.21], *p* < 0.001). Adjusted models: CFUI score was negatively associated with DBP (β = − 0.15 [− 0.31, − 0.002], *p* < 0.001). Stratified by gender, CFUI score was associated with SBP in girls (*p* = 0.018), but not boys (*p* = 0.84). **IQ scores:** Simple model: CFUI score positively associated with total, verbal, and performance IQ scores. Adjusted models: CFUI score positively associated with total IQ (β = 1.92 [1.38, 2.47], *p* < 0.001), verbal IQ (β = 1.92 [1.37, 2.48], *p* < 0.001) and performance IQ (β = 1.33 [0.74, 1.92], *p* < 0.001). **Lipid profile:** No significant association.
Healthy and unhealthy diet score	Jacka et al. (2011) [275]	- Australia - μ11-18y; majority of students <15y (data not shown); 44%F - Data set: It’s Your Move (IYM)	+	**Mental health:** Adjusted models: Healthy diet score positively associated with PedsQL scores (β = 0.29, 95%CI 0.17, 0.43, *p* < 0.001). Unhealthy diet scores negatively associated with PedsQL scores (β = − 0.17, 95%CI − 0.28, − 0.05, *p* < 0.004).
Alternative Health Eating Index (AHEI)	Harris et al. (2016) [244]	- USA - Range 3-18y; 100%F - Dataset: The Nurses’ Health Study II (NHS ll)	+	**Premenopausal breast cancer:** Adjusted models: Top third, fourth and fifth quintiles of diet quality negatively associated with premenopausal breast cancer incidence (HR: 0.78, 95%CI = 0.63,0.97; HR: 0.86, 95%CI = 0.69,1.07; HR: 0.84, 95%CI: 0.67,1.04 respectively). **Postmenopausal breast cancer:** No significant association
Nutrition Quality Index (NQI) and Revised Children’s Diet Quality Index (RC-DQI)	Cheng et al. (2010) [117]	- Germany - Baseline: μ7.4y(SD: 1.3y), Age of onset of pubertal growth spurt: μ9.4y(SD: 1.2y); 53.6% F - Data set: The DONALD Study	+	**Anthropometry:** Simple models: NQI score positively associated with FFMI Z-score (− 0.2 95%CI − 0.4, 0.1, p = 0.04). RC-DQI negatively associated with BMI Z-score (− 0.1 95%CI − 0.3, 0.2, *p* = 0.048) and FMI Z-score (− 0.1 95%CI − 0.3, 0.2, p = 0.04) at the onset of puberty growth spurt. Adjusted models: no significant association. **Timing of puberty:** Adjusted models: scores positively associated with timing of puberty (9.2, 95% CI 9.0, 9.4, p = 0.02). RC-DQI score not associated with the onset of pubertal growth spurt.
Modified Healthy Eating Index (mHEI)	Hooshmand et al. (2018) [149]	- Iran - Baseline: μ13.6y(SD: 3.7y);57%F - Dataset: the Tehran Lipid and Glucose Study (TLGS) Baseline (1999–2001), surveys II (2002–2005), III (2006–2008), and IV (2009–2011).	+	**MetS:** Simple models: mHEI score negatively associated with MetS incidence (OR: 0.38, 95%CI 0.14,1.04). Adjusted models: mHEI score negatively associated with MetS incidence (OR: 0.35, 95%CI 0.13, 0.98).
Modified Mediterranean Diet Quality Index for children and adolescents (m-KM)	Martin-Calvo et al. (2016) [254]	- USA - Range: 8-15y; 55%F - Dataset: The ongoing Growing Up Today Study (GUTS) II cohort (est. 2004, follow up: 2006, 2008, 2011)	+	**Anthropometry:** Simple model: m-KM score was negatively associated with BMI (β = − 0.04 95%CI − 0.07, 0.02, p = 0.001). Adjusted model: m-KM score was negatively associated with BMI (*p* < 0.001).
Raine Eating Assessment in Toddlers (EAT) score	Meyerkort et al. (2012) [38]	- Australia - Range: mid gestation-17y (follow up at: 3, 5, 8, 10, 14 or 17y); 49%F - Dataset: The Western Australian Pregnancy Cohort (Raine) Study (1989–1991)	+	**Anthropometry:** Simple models: The EAT score at 1y associated with BMI at 5y (*p* = 0.009), 8y (p = 0.003), 10y (p = 0.001), 14y (p = 0.001) and 17y (*p* < 0.001). Adjusted models: EAT score at 1y associated with BMI at 5y (*p* = 0.025), 8y (*p* = 0.019), 10y (*p* = 0.013).
Unnamed dietary score	Okubo et al. (2015) [85]	- UK - 6 m, 12 m, 3y & 6y; 44.9%F - Dataset: The Southampton Women’s Survey study	+	**Anthropometry:** Simple models: score associated with fat mass at 6 yrs. (*P* < 0.001). Adjusted models: score negatively associated with fat mass at 6 yrs. (*P* = 0.01).
Diet Quality Index International (DQI-I)	Setayeshgar et al. (2017) [252]	- Canada - Range: 8-10y Baseline: μ9.6(SD: 0.9) Follow up: μ 11.6(SD: 0.9); 45%F - Dataset: QUALITY (QUebec Adipose and Lifestyle InvesTigation in Youth) study	+	**Anthropometry:** Adjusted models: DQI-I score was negatively associated with lower gain in CFMI (β = − 0·08; 95% CI − 0·17, − 0·003) and %BF (β = − 0·55; 95% CI −1·08, − 0·02).

*BF* Body fat, *BMI* Body mass index, *BP* Blood pressure, *Ca* Calcium, *CBF* Central body fat, *CFMI* Central fat mass index, *CI* Confidence interval, *Fe* iron, *FFMI* Fat free mass index, *FFQ* Food frequency questionnaire, *FMI* Fat mass index, *FPG* Fasting plasma glucose, *Hb* Haemoglobin, *HDL* High-density lipoprotein, *HDQ* High diet quality, *HR* Hazzard ratio, *IQR* Interquartile range, *LDL* Low-density lipoprotein, *m* months, *mCHG* mean corpuscular haemoglobin, *MetS* Metabolic Syndrome, *MDP* Mediterranean dietary pattern, *Min., max* minimum and maximum for continuous variables, *OR* Odds ratio, *PedsQL* Pediatric Quality of Life Inventory, *SBP* Systolic blood pressure, *SD* Standard deviation, *SDQ* Strength and difficulties questionnaire, *SEIFA* Socio-economic Indexes for Areas, *TC* Total cholesterol, *TG* Triglycerides, *TIDM* Type 1 diabetes mellitus, *TIIDM* Type 2 diabetes mellitus, *WC* Waist circumference, *Zn* Zinc

### The validation of diet quality indices

Only 28% (*n* = 37) of the DQIs identified were evaluated for validity (*n* = 35) and/or reliability (*n* = 11) (Table 3). Validity was assessed by construct validity (*n* = 21), concurrent or convergent validity (*n* = 8), relative validity (*n* = 8), content validity (*n* = 4), predictive validity (*n* = 4), or comparative validity (*n* = 1), and eight DQIs were assessed for more than one type of validity [46, 107, 152, 177, 192, 240, 268, 272]. Reference standards used to evaluate the validity of indices were other validated tools, serum biomarkers (*n* = 9) [45, 50, 74, 80, 88, 130, 194, 265, 266], food intake (*n* = 18) [19, 33, 45, 69, 73, 74, 80, 87, 88, 107, 121, 130, 152, 172, 175, 177, 178, 265, 266], nutrient intake (*n* = 30) [19, 33, 43, 45, 46, 53, 69, 73, 74, 80, 87, 88, 104, 107, 121, 130, 151, 152, 172, 175, 177, 178, 189, 192, 194, 240, 265, 266, 270–272] and energy intake (*n* = 9) [43, 69, 73, 104, 107, 172, 194, 200, 240, 272]. Cross-sectional health markers including blood pressure (*n* = 1) [80], weight (*n* = 3) [45, 87, 122], BMI (*n* = 11) [33, 45, 73, 80, 87, 121, 122, 200, 240, 270, 271], and waist circumference (*n* = 1) [271], percent body fat (*n* = 2) [122, 270] were used to evaluate validity (Table 3). Although assessed, the Modified revised children’s diet quality index (M-RCDQI) [151] and the Revised Brazilian Healthy Eating Index (BHEI-R) [266] were found to require further research to test the validity and reliability of these tools before they could be considered valid or reliable.

### Health-related outcomes

Only 12 DQIs were evaluated for association with prospective health outcomes (*n* = 12 studies). Measured outcomes from these 12 studies included nutrient biomarkers (*n* = 7) [74, 88, 269, 273], IQ scores (*n* = 1) [269], blood pressure (*n* = 2) [269, 273], plasma cholesterol (*n* = 2) [269, 273], risk of metabolic syndrome (*n* = 1) [149], mental health (*n* = 1) [275], pre and post-menopausal breast cancer (*n* = 1) [244], and timing of puberty (*n* = 1) [117] (Table 4). Anthropometric values examined included BMI (*n* = 7) [38, 74, 85, 88, 252, 254, 269], changes in BMI or fat mass (*n* = 2) [117, 252], changes in weight (*n* = 1) [74], and body composition at onset of puberty (*n* = 1) [117].

Significant associations were found between high diet quality and serum vitamin D (β = 0.005, 95% CI = 0·002, 0·008, *p* < 0.0001), holo-transcobalamin (an indicator of B12) (β = 1·005, 95% CI = 1·002, 1·007, *p* = 0.0002), n-3 FS status (β = 0·376, 95% CI = 0·105, 0·646, *p* < 0·007) [88], and serum vitamin A (r = 0.128, *p* = 0.004) [74]. In adjusted models there were significant positive associations between CFUI score and total IQ (β = 1.92 [1.38, 2.47], *p* < 0.001), verbal IQ (β = 1.92 [1.37, 2.48], *p* < 0.001), and performance IQ (β = 1.33 [0.74, 1.92], *p* < 0.001) [269].

In adjusted models, significant inverse associations were found between diet quality and waist circumference (β = −0.15 [−0.31, − 0.002], *p* = 0.046), diastolic blood pressure (β = − 0.15 [− 0.31, − 0.002], *p* < 0.001) [269] and incidence of metabolic syndrome (OR: 0.35, 95%CI = 0.13,0.98, *p* < 0.05) [149] (Table 4). Significant inverse associations were found between diet quality and HbA1c levels in youth with type 1 diabetes (β = − 0.2, SE = 0.07, *p* = 0.0063). There was no association between diet quality and HbA1c in youth type 2 diabetes; however, there was a significant association for improved systolic blood pressure (β = −2.02, SE = 0.97, *p* = 0.0406) [273, 274].

Diet quality was positively associated with mental health-related quality of life [275] (Table 4). Female children and adolescents with the top three quintiles of diet quality and followed into adulthood had decreased risk of premenopausal breast cancer (HR: 0.78, 95%CI = 0.63,0.97; HR 0.86, 95%CI = 0.69,1.07; and HR 0.84, 95%CI = 0.67,1.04 respectively); but no association was found between AHEI score and pre- or postmenopausal breast cancer (Table 4) [244].

In addition to the above; three studies used prospective health outcomes to evaluate the predictive validity of DQIs (Table 3). The CFUI was associated with improved BMI, waist circumference, and blood pressure [269]; the E-KINDEX was associated with improved BMI, total body fat, and waist circumference [271]; and the Diet Quality Score for Preschool Children was associated with improved fat-free mass and fat mass [177].

## Discussion

This review summarises 128 unique a-priori DQIs used in children and adolescents internationally; however, only 30% were assessed for validity and reliability, from which two were found to require refinement [151, 266] to achieve suitable accuracy and reliability. Additionally, only 15 DQIs were tested for association with prospective health outcomes; finding associations between high diet quality and improved nutrient status, IQ, body composition, risk of metabolic syndrome, blood pressure, HbA1c, mental-health related quality of life, and premenopausal breast cancer.

This systematic review update identified 81 novel paediatric a-priori DQIs (from 157 publication), a 172% increase over 7 years from the 47 identified in the original systematic review [14]. This steep increase in the development and use of DQIs demonstrates that this approach to assessing diet quality is well-utilised within research in children and adolescents internationally. The USA, Australia, Germany, and Brazil appear to be leading the development of paediatric DQIs, together producing 45% of all paediatric DQIs. Beyond these four countries, the vast majority of other DQIs were from other developed countries, possibly reflecting this review’s eligibility criteria. Dietary assessment in developing countries are often focused on assessing growth in an environment characterised by a high prevalence of undernutrition, and and is assessed using non-a-priori diet diversity indices (DDIs), diet diversity scores (DDSs), and food variety scores (FVSs) [14, 138, 167, 224] of which there were 127 excluded from this review (Fig. 1).

There were significant variations in DQIs methods. Simpler scoring methods awarded and summed points for foods which were or were not consumed over a specific frequency. This simple food-based scoring method reduces burden on both researchers, clinicians, and individual users as they can be easily applied to clinical practice. Food-based DQIs included the KIDMED, DGI-CA and ACARFS [33, 35, 214]. More complex DQI scoring methods involved quantification of nutrient intakes from reported food intakes which then undergoes a further step of calculating nutrient intakes relative to age-specific dietary guidelines or energy intake, which make such scores less applicable to the clinical setting or for individual use [141]. DQIs with complex nutrient-based scoring approaches included the NIS [114] and the NQI [115], with DQIs which used a combination of food and nutrient-based scoring methods being more common, such as the ARFS-P [19] and the DGI [36], which embody the same limitations as nutrient-only scoring methods.

Of concern, only 29% of the 128 unique DQIs identified were evaluated for validity and/or reliability, and only 12% evaluated associations with prospective health outcomes. Of the 35 DQIs which were evaluated for validity, 34 were stated to be validated tools by authors; however, due to inconsistent methodological approaches the validity of the DQIs could not be consistently evaluated. Only five DQIs (5%; DQI-A [88], diet quality score for preschool children [177], CFUI [269], E-KINDEX [75] and HNSP [74]) were both evaluated for validity and found to be positively associated with prospective nutrient biomarkers, blood pressure, IQ, and body composition. This suggests these DQIs are the most rigorous in terms of accuracy, reliability, and relevance to health. While the use of DQIs to measure the diet quality of children and adolescents is a highly utilised assessment method, further research is required to address the current paucity of evaluation studies of currently available tools.

Further, the large number of new yet non-validated paediatric a-priori DQIs suggests new DQIs are developed prior to evaluating existing DQIs, and therefore may have been unnecessary. The use of DQIs which have not been rigorously developed and evaluated may compromise the research in which they were used and lead to inaccurate and/or unreliable results. This is particularly the case for DQIs which were developed specifically to evaluate outcomes of a particular study, where the development of the tool was minimally described and not intended for re-use or replication; therefore, limiting confidence in the study results.

Approximately half of the identified DQIs were modified forms of the DQI or HEI [227, 250]. However, only 16 of these modified DQIs were validated in the new population (e.g. age, culture, country) group, where the remaining studies assumed validity based upon the tool being valid in the original population. Non-validated tools, even if adapted from a valid tool, should be used with caution as the modified DQI may not accurately assess diet quality or be appropriately extrapolated to the diet and cultural context of the new population sample. This is particularly the case for modified DQIs in which the scoring system was still based on national dietary guidelines of the original country (e.g. The USA), and not the new population (e.g. Brazil, Canada) [50, 59, 70]. Similar cautions should apply for DQIs such as the Healthy Diet Indicator and the Alternative Healthy Eating Index used in paediatric populations that were designed for adults as these indices may not accurately assess children and adolescent’s diet quality [89, 245].

A factor that varied between papers was the method of dietary data collection, with some DQIs able to be calculated using a variety of dietary assessment methods such as the Diet Quality Index – International [252]. This variety is a strength as it allows flexibility in the application of DQIs in future research and clinical practice. A 24-h recall was the most frequently used dietary assessment tool; however, it is unclear if the 24-h recalls were repeated over several days to improve its accuracy in reporting usual intake. Although most remaining DQIs used FFQs, a substantial number of papers did not use validated methods to collect dietary data [39]. There should also be a caution for the use of single 24-h recalls in studies with small sample sizes or in clinical practice as this one-off measure does not accurately represent usual dietary intake. Although a DQI may be valid, the method of dietary intake assessment must also be accurate and relevant if results are to be interpreted with confidence.

### Limitations and future directions

The present review may be limited by publication bias, particularly in the fields of a null or negative result relating to the validity of DQIs and their association with health-related outcomes; however, publication bias was unable to be assessed as funnel plots were not able to be generated. Although this review reported validity, reliability, and associations with health-related outcomes; it did not evaluate other aspects of assessment tool utility such as sensitivity to change and participant burden nor did it evaluate the validity and reliability of dietary intake assessment methods.

Limitations in the existing literature highlight the need for future research to validate existing paediatric a-priori DQIs and to test their associations with prospective health-related outcomes. This will allow determination of the effect of diet quality during childhood and adolescence on physical health, mental health, and growth which is of increasing importance as the prevalence of diet-related NCDs continues to rise. The application of any DQI should appropriately assess dietary intake using validated methodology and researchers developing new DQIs should ensure that tools reflect indicators of alignment with an appropriate national dietary guideline or nutrient target specific to the culture, country, and age-group of the intended population, and rigorously describe the tools development, scoring method, and validation procedures. Researchers should consider applying existing valid DQIs to their data and undertaking reliability and validity studies in their population groups. For research reporting associations with health-related outcomes, researchers should fully describe the demographic and medical characteristics of the sample, information about dataset used, and transparently detail the results.

### Implications for practice

DQIs present an important opportunity to measure the quality of the total diet of individuals and groups. The current review can be used as a resource to assist health professionals in identifying relevant and valid DQIs for their clinical setting. When selecting a DQI, health professionals should consider: i) whether the DQI demonstrated validity and/or reliability, ii) does the DQI reflect a nutritional reference standard which is relevant to the population in which it will be applied, iii) can the DQI be easily calculated in the clinical setting, and finally iv) can the DQI be calculated by a dietary assessment method which can be performed efficiently in the clinical setting? Although it would be ideal to select a DQI which is associated with prospective health outcomes; due to the paucity of research in this area, this is not yet a feasible consideration*.*

## Conclusion

Research examining diet quality among children and adolescents is of increasing interest globally. However, few indices have been evaluated for validity or reliability or examined for a relationship with prospective health outcomes. Rigorously developed DQIs which have been evaluated have shown good validity, reliability, and association with a range of physical and mental health outcomes. Longitudinal studies are needed to determine the ability of diet quality indices to predict optimal growth and diet-related health-related outcomes among children and adolescents.

## Data Availability

Not applicable.

## Appendix

**Table 5:** Full systematic search strategy and search results implemented across five electronic databases

DATABASE	SEARCH TERMS	HITS
PubMed	(((DQI[Title/Abstract] OR “Healthy Eating Index”[Title/Abstract] OR HEI[Title/Abstract] OR YHEI[Title/Abstract] OR “Recommended Food Score”[Title/Abstract] OR RFS[Title/Abstract] OR “variety score”[Title/Abstract] OR “variety index”[Title/Abstract] OR “variety indices”[Title/Abstract] OR “diversity score”[Title/Abstract] OR “diversity index”[Title/Abstract] OR “diversity indices”[Title/Abstract] OR DDI[Title/Abstract] OR ACARFS[Title/Abstract] OR KIDMED[Title/Abstract] OR “DGI CA”[Title/Abstract] OR FVI[Title/Abstract] OR KINDEX[Title/Abstract] OR AMQI[Title/Abstract] OR “diet quality”[Title/Abstract] OR “dietary quality”[Title/Abstract] OR “diet quality”[Title/Abstract] OR “dietary variety”[Title/Abstract]))) AND ((Child*[Title/Abstract] OR child[Mesh] OR infant*[Title/Abstract] OR infant[Mesh] OR toddler*[Title/Abstract] OR adolescent*[Title/Abstract] OR adolescent[Mesh] OR minor*[Title/Abstract] OR minors[Mesh] OR youth*[Title/Abstract] OR teen*[Title/Abstract] OR pre-teen[Title/Abstract] OR kid*[Title/Abstract])) Search dates: 31 October 2013–10 January 2019	2076
CINAHL	(MH “Child+”) OR (MH “infant+”) OR (MH “adolescent+”) OR (MH “minors+”) OR TI Child* OR TI infant* OR TI toddler* OR TI adolescent* OR TI minor* OR TI youth* OR TI teen* OR TI pre-teen OR TI kid* OR AB Child* OR AB infant* OR AB toddler* OR AB adolescent* OR AB minor* OR AB pre-teen AB kid* AND TI DQI OR TI DQI-I OR TI “Healthy Eating Index” OR TI HEI OR TI YHEI OR TI “Recommended Food Score” OR TI RFS OR TI “variety score” OR TI “variety ind*” OR TI “diversity score” OR TI “diversity ind*” OR TI DDI OR TI ACARFS OR TI KIDMED OR TI “DGI CA” OR TI FVI OR TI KINDEX OR TI AMQI OR TI “diet* quality” OR TI “dietary variety” OR AB DQI OR AB DQI-I OR AB “Healthy Eating Index” OR AB HEI OR AB YHEI OR AB “Recommended Food Score” OR AB RFS OR AB “variety score” OR AB “variety ind*” OR AB “diversity score” OR AB “diversity ind*” OR AB DDI OR AB ACARFS OR AB KIDMED OR AB “DGI CA” OR AB FVI OR AB KINDEX OR AB AMQI OR AB “diet* quality” OR AB “dietary variety” Search dates: 31 October 2013–10 January 2019	814
Embase	‘Child’/exp. OR ‘infant’/exp. OR ‘adolescent’/exp. OR ‘minor (person)’/exp. AND (Child*:ab,ti OR infant*:ab,ti OR toddler*:ab,ti OR adolescent*:ab,ti OR minor*:ab,ti OR youth*:ab,ti OR teen*:ab,ti OR pre-teen:ab,ti OR kid*:ab,ti) AND (DQI:ab,ti OR DQI-I:ab,ti OR “Healthy Eating Index”:ab,ti OR HEI:ab,ti OR YHEI:ab,ti OR “Recommended Food Score”:ab,ti OR RFS:ab,ti OR “variety score”:ab,ti OR “variety ind*”:ab,ti OR “diversity score”:ab,ti OR “diversity ind*”:ab,ti OR DDI:ab,ti OR ACARFS:ab,ti OR KIDMED:ab,ti OR DGI CA:ab,ti OR FVI:ab,ti OR E-KINDEX:ab,ti OR AMQI:ab,ti OR “diet quality”:ab,ti OR “dietary quality”:ab,ti OR “dietary variety”:ab,ti) From inception– 11 January 2019	4754
CENTRAL	((Child*:ti,ab OR [mh child] OR infant*:ti,ab OR [mh infant] OR toddler*:ti,ab OR adolescent*:ti,ab OR [mh adolescent] OR minor*:ti,ab OR [mh minors] OR youth*:ti,ab OR teen*:ti,ab OR pre-teen:ti,ab OR kid*:ti,ab)) AND ((DQI:ti,ab OR DQI-I:ti,ab OR “Healthy Eating Index”:ti,ab OR HEI:ti,ab OR YHEI:ti,ab OR “Recommended Food Score”:ti,ab OR RFS:ti,ab OR “variety score”:ti,ab OR “variety ind*”:ti,ab OR “diversity score”:ti,ab OR “diversity ind*”:ti,ab OR DDI:ti,ab OR ACARFS:ti,ab OR KIDMED:ti,ab OR DGI CA:ti,ab OR FVI:ti,ab OR KINDEX:ti,ab OR AMQI:ti,ab OR “diet quality”:ti,ab OR “dietary quality”:ti,ab OR “dietary variety”:ti,ab)) From inception– 11 January 2019	4329 (trials)
Web of Science	(Child* OR child OR infant* OR infant OR toddler* OR adolescent* OR adolescent OR minor* OR minors OR youth* OR teen* OR pre-teen OR kid*) AND (DQI OR DQI-I OR “Healthy Eating Index” OR HEI OR YHEI OR “Recommended Food Score” OR RFS OR “variety score” OR “variety ind*” OR “diversity score” OR “diversity ind*” OR DDI OR ACARFS OR KIDMED OR DGI CA OR FVI OR KINDEX OR AMQI OR “diet quality” OR “dietary quality” OR “diet variety”) From inception– 11 January 2019	3581

## Abbreviations

CVD: Cardiovascular disease
DDIs: Diet diversity indices
DDSs: Diet diversity scores
DQIs: Diet quality indices
DQI: The diet quality index (a version of diet quality indices)
EDNP: Energy-dense, nutrient-poor foods
FFQ: Food frequency questionnaire
FVSs: Food variety scores
HEI: Healthy eating index
IQ: Intelligence quotient
NCD: Non-communicable diseases
QCC: Quality criteria checklist
T2DM: Type 2 diabetes mellitus

## References

[1] Torpy JM, Campbell A, Glass RM (2010). Chronic diseases of children. JAMA.

[2] Australian Institute of Health and Welfare (2005). Selected Chronic Diseases Among Australia’s Children. AIHW cat no AUS 62.

[3] Diabetes Australia (2015). Type 2 diabetes in younger people: small but significant.

[4] Larkins NG, Teixeira-Pinto A, Craig JC (2018). The prevalence and predictors of hypertension in a National Survey of Australian Children. Blood Pressure.

[5] Riley M, Bluhm B (2012). High blood pressure in children and adolescents. Am Fam Phys.

[6] Falkner B (2010). Hypertension in children and adolescents: epidemiology and natural history. Pediatr Nephrol.

[7] Michaud PA, Suris JC, Viner R. The Adolescent with a Chronic Condition: Epidemiology, developmental issues and health care provision. Geneva: World Health Organisation; 2007.

[8] World Health Organisation (2017). New global estimates of child and adolescent obesity released on World Obesity Day.

[9] NCD Child (2018). Understanding NCDs.

[10] The NCD Alliance (2011). A Focus on Children and Non-Communicable Diseases (NCDs).

[11] Green R, Sutherland J, Dangour AD, Shankar B, Webb P (2016). Global dietary quality, undernutrition and non-communicable disease: a longitudinal modelling study. BMJ Open.

[12] Wirt A, Collins CE (2009). Diet quality – what is it and does it matter?. Public Health Nutr.

[13] National Health and Medical Research Council (2017). What are Nutrient Reference Values?.

[14] Marshall S, Burrows T, Collins C (2014). Systematic review of diet quality indices and their associations with health-related outcomes in children and adolescents. J Hum Nutr Diet.

[15] Australian Bureau of Statistics (2017). Children’s risk factors.

[16] Al-Khudairy L, Loveman E, Colquitt J, Mead E, Johnson R, Fraser H, et al. Diet, physical activity and behavioural interventions for the treatment of overweight or obese adolescents aged 12 to 17 years. Cochrane Database Syst Rev. 2017;(6) Available from. 10.1002/14651858.CD012691. 10.1002/14651858.CD012691 PMC6481371 28639320

[17] Morgan PJ, Collins CE, Plotnikoff RC, McElduff P, Burrows T, Warren JM (2010). The SHED-IT community trial study protocol: a randomised controlled trial of weight loss programs for overweight and obese men. BMC Public Health.

[18] Duncanson K, Lee YQ, Burrows T, Collins C (2017). Utility of a brief index to measure diet quality of Australian preschoolers in the Feeding Healthy Food to Kids Randomised Controlled Trial. Nutr Diet.

[19] Burrows TL, Collins K, Watson J, Guest M, Boggess MM, Neve M (2014). Validity of the Australian Recommended Food Score as a diet quality index for Pre-schoolers. Nutrit J.

[20] Robinson LN, Rollo ME, Watson J, Burrows TL, Collins CE (2015). Relationships between dietary intakes of children and their parents: a cross-sectional, secondary analysis of families participating in the Family Diet Quality Study. J Hum Nutr Diet.

[21] Moher D, Liberati A, Tetzlaff J, Altman DG (2010). Preferred reporting items for systematic reviews and meta-analyses: The PRISMA statement. Int J Surg.

[22] Centre for Research in Evidence Based Practice (2017). Polyglot search.

[23] Google Translate (2017). Google Translate.

[24] Rathbone J, Carter M, Hoffmann T, Glasziou P (2015). Better duplicate detection for systematic reviewers: evaluation of Systematic Review Assistant-Deduplication Module. Systematic reviews..

[25] Clarivate Analytics (2018). Endnote.

[26] Covidence. Covidence. Melbourne: Veritas Health Innovation. Accessed Sept 2018.

[27] American Dietetic Association. Evidence analysis manual: steps in the ada evidence analysis process. Chicago: Academy of Nutrition & Dietetics; 2010. Contract No.: ISBN: 978–0–88091-429-1.

[28] Tonkin E, Kennedy D, Golley R, Byrne R, Rohit A, Kearns T (2018). The Relative Validity of the Menzies Remote Short-Item Dietary Assessment Tool (MRSDAT) in Aboriginal Australian Children Aged 6–36 Months. Nutrients..

[29] Australian Government (2013). Australian Dietary Guidelines.

[30] Rohit A, Brimblecombe J, O’ Dea K, Tonkin E, Maypilama Ḻ, Maple-Brown L (2018). Development of a short-item diet quality questionnaire for Indigenous mothers and their young children: The Menzies remote short-item dietary assessment tool. Aust J Rural Health..

[31] Bell LK, Golley RK, Magarey AM. A short food-group-based dietary questionnaire is reliable and valid for assessing toddlers’ dietary risk in relatively advantaged samples – Corrigendum. 2014;112(9):1587-. 10.1017/S0007114514001184 24886781

[32] Russell CG, Worsley A (2007). Do children’s food preferences align with dietary recommendations?. Public Health Nutr.

[33] Marshall S, Watson J, Burrows T, Guest M, Collins CE (2012). The development and evaluation of the Australian child and adolescent recommended food score: a cross-sectional study. Nutr J.

[34] Gasser CE, Kerr JA, Mensah FK, Wake M (2017). Stability and change in dietary scores and patterns across six waves of the Longitudinal Study of Australian Children. Br J Nutr.

[35] Golley R, Hendrie G, McNaughton S (2011). The Dietary Guidelines Index for Children and Adolescents (DGI-CA).

[36] Lioret S, McNaughton SA, Cameron AJ, Crawford D, Campbell KJ, Cleland VJ (2014). Three-year change in diet quality and associated changes in BMI among schoolchildren living in socio-economically disadvantaged neighbourhoods. Br J Nutr.

[37] Jacka F, Kremer P, Leslie E, Berk M, Patton G, Toumbourou JW (2010). Associations between diet quality and depressed mood in adolescents: results from the Australian Healthy Neighbourhoods Study. Aust N Z J Psych..

[38] Meyerkort CE, Oddy WH, O'Sullivan TA, Henderson J, Pennell CE (2012). Early diet quality in a longitudinal study of Australian children: associations with nutrition and body mass index later in childhood and adolescence. J Devel Orig Health Dis..

[39] Nyaradi A, Oddy WH, Hickling S, Li J, Foster JK (2015). The relationship between nutrition in infancy and cognitive performance during adolescence. Front Nutr.

[40] Li J, O'Sullivan T, Johnson S, Stanley F, Oddy W (2012). Maternal work hours in early to middle childhood link to later adolescent diet quality. Public Health Nutr..

[41] National Health and Medical Resarch Council (2005). Nutrient Reference Values for Australia and New Zealand Including Recommended Dietary Intakes.

[42] Scott JA, Chih TY, Oddy WH (2012). Food variety at 2 years of age is related to duration of breastfeeding. Nutrients..

[43] Spence AC, McNaughton SA, Lioret S, Hesketh KD, Crawford DA, Campbell KJ (2013). A health promotion intervention can affect diet quality in early childhood. J Nutr..

[44] Dietary Guidelines for Americans 2015–2020 Eigth Edition. In: Services UDoHaH, editor. dietaryguidelines.gov: USDA; 2015.

[45] Kunaratnam K, Halaki M, Wen LM, Baur LA, Flood VM (2018). Reliability and comparative validity of a Diet Quality Index for assessing dietary patterns of preschool-aged children in Sydney, Australia. Eur J Clin Nutr.

[46] Huybrechts I, Vereecken C, De Bacquer D, Vandevijvere S, Van Oyen H, Maes L (2010). Reproducibility and validity of a diet quality index for children assessed using a FFQ. Br J Nutr.

[47] Vlaams Instituut voor Gezondheidspromotie (VIG) (2004). voedingsdriehoek: een Praktische Voedingsgids: VIG.

[48] Sabbe D, De Bourdeaudhuij I, Legiest E, Maes L (2008). A cluster-analytical approach towards physical activity and eating habits among 10-year-old children. Health Educ Res.

[49] Dietary Guideline Advisory Committee. Report of the Dietary Guidelines Advisory Committee for Americans, 2000 (2000). To the Secretary of Health and Human Services and Secretary of Agriculture.

[50] Rauber F, da Costa Louzada ML, Vitolo MR (2014). Healthy eating index measures diet quality of Brazilian children of low socioeconomic status. J Am College Nutr.

[51] Vítolo MR (2002). Dez passos para uma alimentaçào saudável. Guia alimentar para crianças menores de 2 anos: um guia para o profissional da saúde na atençào básica.

[52] Molina Mdel C, Lopez PM, Faria CP, Cade NV, Zandonade E (2010). Socioeconomic predictors of child diet quality. Revista de saude Publica..

[53] Conceição SIOD, Oliveira BR, Rizzin M, Silva AAMD (2018). Healthy Eating Index: adaptation for children aged 1 to 2 years. Ciencia Saude Coletiva.

[54] Fisberg RM, Slater B, Barros RR, De Lima FD, Cesar CLG, Carandina L (2004). Índice de Qualidade da Dieta: Avaliação da adaptação e aplicabilidade. Healthy Eating Index Eval Adapted Version Appl.

[55] Philippi ST, Latterza AR, Cruz ATR, Ribeiro LC (1999). Pirâmide alimentar adaptada: guia para escolha dos alimentos. Revista de nutrição..

[56] Previdelli AN, Andrade SC, Pires MM, Ferreira SRG, Fisberg RM, Marchioni DM (2011). A revised version of the Healthy Eating Index for the Brazilian population. Revista de saude Publica..

[57] Paulo Rogério Melo R, Gomes de S RA, Mara Lima De C, Luana Silva M, Camila Pinheiro C, Alessandra Page B (2016). Dietary quality varies according to data collection instrument: a comparison between a food frequency questionnaire and 24-hour recall. Cad Saúde Pública.

[58] Simone C, Semíramis Martins Álvares D (2013). Diet quality index for healthy food choices. Rev Nutrição..

[59] Juliana Garcia B, Andréa Polo G, Aline De Piano G (2016). Impact of actions of food and nutrition education program in a population of adolescents. Revista de Nutrição..

[60] Wendpap LL, Ferreira MG, Rodrigues PRM, Pereira RA, Loureiro ADS, Gonçalves-Silva RMV (2014). Adolescents’ diet quality and associated factors. Cad Saude Publica.

[61] Jessri M, Ng A, L’Abbé M (2017). Adapting the Healthy Eating Index 2010 for the Canadian Population: Evidence from the Canadian Community Health Survey. Nutrients..

[62] Nshimyumukiza L, Lieffers JR, Ekwaru JP, Ohinmaa A, Veugelers PJ (2018). Temporal changes in diet quality and the associated economic burden in Canada. PloS one..

[63] Bush MA, Martineau C, Pronk JA, Brulé D (2007). Eating well with Canada's food guide:“A tool for the times”. Can J Diet Pract Res..

[64] Glanville NT, Mcintyre L (2006). Diet quality of Atlantic families headed by single mothers. Can J Diet Pract Res.

[65] Health and Welfare Canada and Ontario Ministry of Health (1993). Canada’s Food Guide to Healthy Eating.

[66] Woodruff SJ, Hanning RM (2010). Development and implications of a revised Canadian Healthy Eating Index (HEIC-2009). Public Health Nutr.

[67] Wang JW, Shang L, Light K, O'Loughlin J, Paradis G, Gray-Donald K (2015). Associations between added sugar (solid vs. liquid) intakes, diet quality, and adiposity indicators in Canadian children. Appl Physiol Nutr Metab..

[68] Tugault-Lafleur CN, Black JL, Barr SI (2017). Examining school-day dietary intakes among Canadian children. Appl Physiol Nutr Metabol.

[69] Absolon JS, Wearring GA, Behme MT (1988). Dietary quality and eating patterns of adolescent girls in southwestern ontario. J Nutr Educ Behav.

[70] Protudjer JLP, Sevenhuysen GP, Ramsey CD, Kozyrskyj AL, Becker AB (2012). Low vegetable intake is associated with allergic asthma and moderate-to-severe airway hyperresponsiveness. Pediatr Pulmonol.

[71] Ya-Qun Y, Fan L, Rui-Hua D, Jing-Si C, Geng-Sheng H, Shu-Guang L (2017). The Development of a Chinese Healthy Eating Index and Its Application in the General Population. Nutrients..

[72] Ge K (2011). The transition of Chinese dietary guidelines and the food guide pagoda. Asia Pac J Clin Nutr..

[73] Cheng G, Duan R, Kranz S, Libuda L, Zhang L (2016). Development of a dietary index to assess overall diet quality for chinese school-aged children: the chinese children dietary index. J Acad Nutr Diet.

[74] Peng R, Wei XP, Liang XH, Yang T, Xu JP, Liu YX (2015). Study on dietary screening model for preschool children with vitamin A deficiency in Ba'nan District of Chongqing. J Shanghai Jiaotong Univ (Medical Science)..

[75] Lazarou C, Panagiotakos DB, Matalas AL (2009). Foods E-KINDEX: a dietary index associated with reduced blood pressure levels among young children: the CYKIDS study. J Am Diet Assoc.

[76] Willett WC, Sacks F, Trichopoulou A, Drescher G, Ferro-Luzzi A, Helsing E (1995). Mediterranean diet pyramid: a cultural model for healthy eating. Am J Clin Nutr.

[77] Knudsen V, Fagt S, Trolle E, Matthiessen J, Groth M, Biltoft-Jensen A (2012). Evaluation of dietary intake in Danish adults by means of an index based on food-based dietary guidelines. Food Nutr Res.

[78] Rohde JF, Larsen SC, Ängquist L, Olsen NJ, Stougaard M, Mortensen EL (2017). Effects of the Healthy Start randomized intervention on dietary intake among obesity-prone normal-weight children. Public Health Nutr.

[79] Astrup A, Andersen NL, Stender S, Trolle E (2005). Kostrådene 2005.

[80] Golley RK, Smithers LG, Mittinty MN, Brazionis L, Emmett P, Northstone K (2012). An index measuring adherence to complementary feeding guidelines has convergent validity as a measure of infant diet quality. J Nutr.

[81] National Health and Medical Research Council (2012). Infant Feeding Guidelines Canberra.

[82] Ministry of Health (2008). Food and Nutrition Guidelines for Healthy Infants and Toddlers (Aged 0–2): A background paper Wellington.

[83] Centers for disease control and prevention. Division of Nutrition, Physical Activity, and Obesity, National Center for Chronic Disease Prevention and Health Promotion. Foods and Drinks for 6 to 24 Month Olds: U.S. Department of Health & Human Services; 2018. Available from: https://www.cdc.gov/nutrition/infantandtoddlernutrition/foods-and-drinks/index.html.

[84] Scientific Advisory Committee on Nutrition (2018). Feeding in the first year of life.

[85] Okubo H, Crozier SR, Harvey NC, Godfrey KM, Inskip HM, Cooper C (2015). Diet quality across early childhood and adiposity at 6 years: the Southampton Women's Survey. Int J Obes (2005)..

[86] Ministry of Health LaW (2005). Japanese food guide spinning top.

[87] Rice N, Gibbons H, McNulty BA, Walton J, Flynn A, Gibney MJ (2015). Development and validation testing of a short nutrition questionnaire to identify dietary risk factors in preschoolers aged 12–36 months. Food & nutrition research..

[88] Vyncke KE, Huybrechts I, Dallongeville J, Mouratidou T, Van Winckel MA, Cuenca-García M (2013). Intake and serum profile of fatty acids are weakly correlated with global dietary quality in European adolescents. Nutrition.

[89] Huijbregts P, Feskens E, Räsänen L, Fidanza NF, Nissinen A, Menotti A (1997). Dietary pattern and 20 year mortality in elderly men in Finland, Italy, and The Netherlands: longitudinal cohort study. BMJ.

[90] World Health Organization (1990). World Health Organisation. Diet, nutrition and prevention of chronic diseases. Report of a WHO Study Group Geneva.

[91] Arvidsson L, Eiben G, Hunsberger M, De Bourdeaudhuij I, Molnar D, Jilani H (2017). Bidirectional associations between psychosocial well-being and adherence to healthy dietary guidelines in European children : prospective findings from the IDEFICS study. BMC Public Health.

[92] Ahrens W, Bammann K, Siani A, Buchecker K, De Henauw S, Iacoviello L (2011). The IDEFICS cohort: design, characteristics and participation in the baseline survey. Int J Obes (2005)..

[93] Oliveira A, Jones L, de Lauzon-Guillain B, Emmett P, Moreira P, Charles MA (2015). Early problematic eating behaviours are associated with lower fruit and vegetable intake and less dietary variety at 4–5 years of age. A prospective analysis of three European birth cohorts. Br J Nutr.

[94] Jones L, Moschonis G, Oliveira A, de Lauzon-Guillain B, Manios Y, Xepapadaki P (2015). The influence of early feeding practices on healthy diet variety score among pre-school children in four European birth cohorts. Public Health Nutr.

[95] De Vriendt T, Clays E, Huybrechts I, De Bourdeaudhuij I, Moreno LA, Patterson E (2012). European adolescents’ level of perceived stress is inversely related to their diet quality: the Healthy Lifestyle in Europe by Nutrition in Adolescence study. Br J Nutr..

[96] Vlaams Instituur voor Gezondheidspromotie (Flemish Institute for Health Promotion and Disease Prevention; VIGeZ) De actieve voedingsdriehoek: een praktische voedings- en beweeggids (The Active Food Guide Pyramid: Stress and diet quality in adolescents 379 British Journal of Nutrition 2008. Available from: https://www.cambridge.org/core.

[97] Lloyd-Jones DM, Hong Y, Labarthe D, Mozaffarian D, Appel L, Van Horn L (2010). Defining and Setting National Goals for Cardiovascular Health Promotion and Disease Reduction The American Heart Association's Strategic Impact Goal Through 2020 and Beyond. Circulation..

[98] Henriksson P, Cuenca-García M, Labayen I, Esteban-Cornejo I, Henriksson H, Kersting M (2017). Diet quality and attention capacity in European adolescents: the Healthy Lifestyle in Europe by Nutrition in Adolescence (HELENA) study. Br J Nutr.

[99] Kavey R-EW, Daniels SR, Lauer RM, Atkins DL, Hayman LL, Taubert K (2003). American heart association guidelines for primary prevention of atherosclerotic cardiovascular disease beginning in childhood. J Pediatr.

[100] Röytiö H, Jaakkola J, Hoppu U, Poussa T, Laitinen K (2015). Development and evaluation of a stand-alone index for the assessment of small children’s diet quality. Public Health Nutr.

[101] Nordic Nutrition recommendations. In: Ministers NCo, editor. Copenhagen: Nord; 2004.

[102] Kanerva N, Kaartinen NE, Schwab U, Lahti-Koski M, Männistö S (2014). The Baltic Sea Diet Score: a tool for assessing healthy eating in Nordic countries. Public Health Nutr.

[103] Haapala E, Eloranta A, Venalainen T, Jalkanen H, Poikkeus A, Ahonen T (2017). Diet quality and academic achievement: a prospective study among primary school children. Eur J Nutr..

[104] Kyttälä P, Erkkola M, Lehtinen-Jacks S, Ovaskainen M-L, Uusitalo L, Veijola R (2014). Finnish Children Healthy Eating Index (FCHEI) and its associations with family and child characteristics in pre-school children. Public Health Nutr.

[105] Guthrie HA, Scheer JC (1981). Validity of a dietary score for assessing nutrient adequacy. J Am Diet Assoc..

[106] Shatenstein B, Abu-Shaaban D, Pascual ML, Kark JD (1996). Dietary adequacy among urban and semi-rural schoolchildren in Gaza. Ecol Food Nutr.

[107] Verger EO, Eussen S, Holmes BA (2016). Evaluation of a nutrient-based diet quality index in UK young children and investigation into the diet quality of consumers of formula and infant foods. Public Health Nutr.

[108] Choices N (2016). The Eatwell Guide.

[109] Schoen S, Jergens S, Barbaresko J, Nöthlings U, Kersting M, Remer T (2017). Diet quality during infancy and early childhood in children with and without risk of type 1 diabetes: A DEDIPAC study. Nutrients.

[110] Department of Health Dietary Reference Values for Food Energy and Nutrients for the United Kingdom. London; 1991.

[111] Alexy U, Kersting M, Schultze-Pawlitschko V (2003). Two approaches to derive a proposal for added sugars intake for German children and adolescents. Public Health Nutr..

[112] Kersting M, Alexy U, Clausen K (2005). Using the concept of food based dietary guidelines to develop an optimized mixed diet (OMD) for German children and adolescents. J Pediatr Gastroenterol Nutr..

[113] Kleiser C, Mensink GBM, Scheidt-Nave C, Kurth BM (2009). HuSKY: A healthy nutrition score based on food intake of children and adolescents in Germany. Br J Nutr.

[114] Alexy U, Sichert-Hellert W, Kersting M, Lausen B, Schoch G (1999). Development of scores to measure the effects of nutrition counselling on the overall diet: A pilot study in children and adolescents. Eur J Nutr.

[115] Gedrich K, Karg G (2001). Dietary habits of German vs Non-German residents in Germany. Culinary Arts and Sciences III - Global and National Perspectives.

[116] Deutsche Gesellschaft für Ernährung (DGE) (2000). Österreichische Gesellschaft für Ernährung (ÖGE), Schweizerische Vereinigung für Ernährung (SGE): Referenzwerte fur die Nahrstoffzufuhr.

[117] Cheng G, Gerlach S, Libuda L, Kranz S, Günther ALB, Karaolis-Danckert N (2010). Diet quality in childhood is prospectively associated with the timing of puberty but not with body composition at puberty onset. J Nutr.

[118] Gedrich K, Karg G (2001). Dietary habits of German vs. non-German residents in Germany.

[119] German Nutrition Society ANS, Swiss Society for Nutrition Research (2002). Reference values for nutrient intake.

[120] Kohlboeck G, Sausenthaler S, Standl M, Koletzko S, Bauer CP, von Berg A (2012). Food intake, diet quality and behavioral problems in children: results from the GINI-plus/LISA-plus studies. Ann Nutr Metabol.

[121] Manios Y, Kourlaba G, Grammatikaki E, Androutsos O, Moschonis G, Roma-Giannikou E (2010). Development of a diet–lifestyle quality index for young children and its relation to obesity: the Preschoolers Diet–Lifestyle Index. Public Health Nutr..

[122] Magriplis E, Farajian P, Risvas G, Panagiotakos D, Zampelas A (2015). Newly derived children-based food index. An index that may detect childhood overweight and obesity. Int J Food Sci Nutr.

[123] U.S. Department of Agriculture (2010). USDA national nutrient database for standard reference.

[124] Yannakoulia M, Karayiannis D, Terzidou M, Kokkevi A, Sidossis LS (2004). Nutrition-related habits of Greek adolescents. Eur J Clin Nutr..

[125] Supreme Scientific Health Council (SSHC) MoHaW (1999). Dietary Guidelines for Adults in Greece.

[126] U.S. Department of Agriculture (USDA) (2000). Report of the Dietary Guidelines Advisory.

[127] Trichopoulou A, Kouris-Blazos A, Wahlqvist ML, Gnardellis C, Lagiou P, Polychronopoulos E (1995). Diet and overall survival in elderly people. Bmj..

[128] Lazarou C, Panagiotakos D, Matalas A-L. E-KINDEX, a novel dietary index that is associated with obesity status in children. Int J Obes. 2008;32.

[129] Lazarou C, Panagiotakos DB, Panayiotou G, Matalas AL (2008). Overweight and obesity in preadolescent children and their parents in Cyprus: prevalence and associated socio-demographic factors – the CYKIDS study. Obes Rev.

[130] Manios Y, Kourlaba G, Grammatikaki E, Koubitski A, Siatitsa P, Vandorou A (2010). Development of a lifestyle–diet quality index for primary schoolchildren and its relation to insulin resistance: the Healthy Lifestyle–Diet Index. Eur J Clin Nutr.

[131] Bach A, Serra-Majem L, Carrasco JL, Roman B, Ngo J, Bertomeu I (2006). The use of indexes evaluating the adherence to the Mediterranean diet in epidemiological studies: a review. Public Health Nutr.

[132] Manios Y, Moschonis G, Papandreou C, Politidou E, Naoumi A, Peppas D (2015). Revised Healthy Lifestyle-Diet Index and associations with obesity and iron deficiency in schoolchildren: The Healthy Growth Study. J Hum Nutr.

[133] U.S Department of Agriculture. USDA Choose My Plate n.d. Available from: https://www.choosemyplate.gov/.

[134] Enneman A, Hernandez L, Campos R, Vossenaar M, Solomons NW (2009). Dietary characteristics of complementary foods offered to Guatemalan infants vary between urban and rural settings. Nutr Res.

[135] Dewey K, Cohen RJ, Arimond M, Ruel MT (2006). Developing and validating simple indicators of complementary food intake and nutrient density for breastfed children in developing countries.

[136] Institute of Nutrition of Central America and Panama. Guías alimentarias para Guatemala: los siete pasos para una alimentación sana. n.d.

[137] Bermudez OI, Hernandez L, Mazariegos M, Solomons NW (2008). Secular trends in food patterns of Guatemalan consumers: new foods for old. Food Nutr Bull.

[138] Chiplonkar SA, Tupe R (2010). Development of a diet quality index with special reference to micronutrient adequacy for adolescent girls consuming a lacto-vegetarian diet. J Am Diet Assoc.

[139] Indian Council of Medical Research (2005). Dietary Guidelines for Indians—A Manual.

[140] Department of Health and Human Services (2005). Dietary Guidelines for Americans. Department of Health and Human Services and U.S. Department of Agriculture, editor.

[141] Prasetyo TJ, Hardinsyah, Sinaga T (2013). Food and Nutrients Intake and Desirable Dietary Pattern Score of Indonesian Children Aged 2–6 Years. Jurnal Gizi Dan Pangan.

[142] Food and Liverstock department of west Java Province. Hope Dietary Pattern Java, Indonesia. n.d. Available from: http://dkpp.jabarprov.go.id/page/Pola-Pangan-Harapan.

[143] Fung T, Chiuve S, McCullough M, Rexrode K, Logroscino G, Hu F (2008). Adherence to a DASH-Style diet and risk of coronary heart disease and stroke in women. Arch Intern Med..

[144] Asghari G, Yuzbashian E, Mirmiran P, Hooshmand F, Najafi R, Azizi F (2016). Dietary Approaches to Stop Hypertension (DASH) Dietary Pattern Is Associated with Reduced Incidence of Metabolic Syndrome in Children and Adolescents. J Pediatr.

[145] National Heart Lung aBI (2006). Your Guide to Lowering Your Blood Pressure With DASH. United States Department of Health and Human Services NIoH, editor.

[146] Mohammad Hossein R, Maryam M, Nasrin O, Ahmad E, Leila A (2012). Fast Food Consumption, Quality of Diet, and Obesity among Isfahanian Adolescent Girls. J Obes.

[147] Thurlow J (2008). Krause's Food and Nutrition Therapy, 12th Edition.

[148] Azadbakht L, Akbari F, Esmaillzadeh A (2015). Diet quality among Iranian adolescents needs improvement. Public Health Nutr.

[149] Hooshmand F, Asghari G, Yuzbashian E, Mahdavi M, Mirmiran P, Azizi F (2018). Modified Healthy Eating Index and Incidence of Metabolic Syndrome in Children and Adolescents: Tehran Lipid and Glucose Study. J Pediatr.

[150] US Department of Agriculture (1992). The Food Guide Pyramid.

[151] Keshani P, Salehi M, Kaveh MH, Faghih S (2018). Self-efficacy and cues to action: Two main predictors of modified version of diet quality index in Iranian adolescents. Progress Nutr.

[152] Kranz S, McCabe GP (2013). Examination of the five comparable component scores of the diet quality indexes HEI-2005 and RC-DQI using a nationally representative sample of 2–18 year old children: NHANES 2003–2006. J Obes.

[153] Fogli-Cawley JJ, Dwyer JT, Saltzman E, McCullough ML, Troy LM, Jacques PF (2006). The 2005 Dietary Guidelines for Americans Adherence Index: development and application. J Nutr.

[154] Mohseni-Takalloo S, Hosseini-Esfahani F, Mirmiran P, Azizi F (2016). Associations of pre-defined dietary patterns with obesity associated phenotypes in Tehranian adolescents. Nutrients.

[155] Perry CP, Keane E, Layte R, Fitzgerald AP, Perry IJ, Harrington JM (2015). The use of a dietary quality score as a predictor of childhood overweight and obesity Chronic Disease epidemiology. BMC Public Health.

[156] Food Safety Authority of Ireland (2011). Scientific Recommendations for Healthy Eating Guidelines in Ireland.

[157] Alkerwi A (2014). Diet quality concept.

[158] Gerber M (2006). Qualitative methods to evaluate Mediterranean diet in adults. Public Health Nutrition..

[159] Tarabusi V, Cavazza C, Pasqui F, Gambineri A, Pasquali R (2010). Quality of diet, screened by the Mediterranean diet quality index and the evaluation of the content of advanced glycation endproducts, in a population of high school students from Emilia Romagna. Mediterranean J Nutr Metabol.

[160] National Research Council Committee on Diet and Health (1989). Diet and Health: Implications for Reducing Chronic Disease Risk. Food and Nutrition Board Commission on Life Sciences.

[161] Food and Nutrition Board (1989). Recommended Dietary Allowances.

[162] Gerber MJ, Scali JD, Michaud A, Durand MD, Astre CM, Dallongeville J (2000). Profiles of a healthful diet and its relationship to biomarkers in a population sample from Mediterranean southern France. J Am Diet Assoc..

[163] Nishimura T, Murakami K, Livingstone MBE, Sasaki S, Uenishi K (2015). Adherence to the food-based Japanese dietary guidelines in relation to metabolic risk factors in young Japanese women. Br J Nutr.

[164] Kuriyama N, Murakami K, Livingstone MBE, Okubo H, Kobayashi S, Suga H (2016). Development of a food-based diet quality score for Japanese: associations of the score with nutrient intakes in young, middle-aged and older Japanese women. J Nutr Sci.

[165] Choi Y, You Y, Go KA, Tserendejid Z, You HJ, Lee JE (2013). The prevalence of obesity and the level of adherence to the Korean Dietary Action Guides in Korean preschool children. Nutr Res Pract.

[166] Ministry of Health and Welfare (2003). The 2003 Dietary Guidelines for Koreans-Dietary Action Guides for Infants & Toddlers: Pregnant & Lactating Women, Children, and Adolescents.

[167] Moursi MM, Arimond M, Dewey KG, Trèche S, Ruel MT, Delpeuch F (2008). Dietary diversity is a good predictor of the micronutrient density of the diet of 6- to 23-month-old children in Madagascar. J Nutr.

[168] Onyango AW (2003). Dietary diversity, child nutrition and health in contemporary African communities. Comp Biochem Physiol A..

[169] FAO/WHO (2002). Vitamin and mineral requirements in human nutrition.

[170] Institute of Medicine (1997). Dietary reference intakes for calcium, phosphorus, magnesium, vitamin D and fluoride.

[171] Institute of Medicine. Dietary reference intakes for vitamin A, vitamin K, arsenic, boron, chromium, copper, iodine, iron, manganese, molybdenum, nickel, vanadium and zinc: National Academy Press; 2003. 25057538

[172] Chen L-W, Fung SM, Fok D, Leong LP, Toh JY, Lim HX (2019). The Development and Evaluation of a Diet Quality Index for Asian Toddlers and Its Perinatal Correlates: The GUSTO Cohort Study. Nutrients..

[173] Health Promotion Board Singapore (2016). A Healthy Food Foundation—For Kids and Teens.

[174] Lee T, Norimah A, Safiah M (2011). Development of Healthy Eating Index for Malaysian adults.

[175] Rezali FW, Chin YS, Shariff ZM, Mohd Yusof BN, Sanker K, Woon FC (2015). Evaluation of diet quality and its associated factors among adolescents in Kuala Lumpur, Malaysia. Nutr Res Pract.

[176] Ministry of Health Malaysia (2013). Malaysian Dietary Guidelines for Children and Adolescents: Summary.

[177] Voortman T, Kiefte-de Jong JC, Geelen A, Villamor E, Moll HA, de Jongste JC (2015). The development of a diet quality score for preschool children and its validation and determinants in the Generation R Study. J Nutr.

[178] van der Velde LA, Nguyen AN, Schoufour JD, Geelen A, Jaddoe VW, Franco OH, et al. Diet quality in childhood: the Generation R Study. Eur J Nutr. 2019;58:1259–69. 10.1007/s00394-018-1651-z PMC6499873 29516225

[179] Health Council of the Netherlands (Gezondheidsraad) (2015). Dutch Guidelines for a Healthy diet.

[180] Netherlands Nutrition Centre (2011). Dutch food-based dietary guidelines (Richtlijnen voedselkeuze).

[181] Kersting MH (2014). Annett. Erna¨hrung bei Kleinkindern: Empfehlungen und Erna¨hrungspraxis [Nutrition in infants: recommendations and nutritional practice]. J für Ernährungsmedizin..

[182] Schweizerische Gesellschaft (2011). fu¨r Erna¨hrung. Erna¨hrung von Kindern [Nutrition of children].

[183] Flemish Institute for Health Promotion and Disease Prevention. De actieve voedingsdriehoek [The Active Food Guide Pyramid]. Brussels; 2012. p. 37.

[184] Public Health Agency. Maternal and pre-school child nutrition guidelines. Belfast; 2012.

[185] US Department of Health and Human Services (2010). Dietary guidelines for Americans.

[186] Skinner AC, Skelton JA (2014). Prevalence and Trends in Obesity and Severe Obesity Among Children in the United States, 1999–2012. JAMA Pediatr.

[187] Cattaneo A, Williams C, Pallás-Alonso CR, Hernández-Aguilar MT, Lasarte-Velillas JJ, Landa-Rivera L (2011). ESPGHAN's 2008 recommendation for early introduction of complementary foods: how good is the evidence?. Matern Child Nutr.

[188] Imhoff-Kunsch B, Briggs V, Goldenberg T, Ramakrishnan U (2012). Effect of n-3 Long-chain Polyunsaturated Fatty Acid Intake during Pregnancy on Maternal, Infant, and Child Health Outcomes: A Systematic Review. Paediatr Perinatal Epidemiol.

[189] Delshad M, Beck KL, von Hurst PR, Mugridge O, Conlon CA (2018). The validity and reliability of the Dietary Index for a Child's Eating (DICE) in 2–8 year old children living in New Zealand. Matern Child Nutr.

[190] NZ Ministry of Health (2012). Food and Nutrition Guidelines for Healthy Children and Young People (Aged 2–18 Years).

[191] NZ Ministry of Health (2005). Nutrient reference values for Australia and New Zealand, (including recommended dietary intakes).

[192] Wong J, Parnell W, Howe A, Black K, Skidmore P. Development and validation of a food-based diet quality index for New Zealand adolescents. BMC Public Health. 2013;13(1). 10.1186/1471-2458-13-562 PMC3706237 23759064

[193] NZ Ministry of Health (1998). Food and Nutrition Guidelines for Healthy Adolescents: A background paper.

[194] Wong JE, Skidmore PML, Williams SM, Parnell WR (2014). Healthy dietary habits score as an indicator of diet quality in New Zealand adolescents. J Nutr.

[195] Handeland K, Kjellevold M, Wik Markhus M, Eide Graff I, Frøyland L, Lie Ø (2016). A Diet Score Assessing Norwegian Adolescents’ Adherence to Dietary Recommendations-Development and Test-Retest Reproducibility of the Score. Nutrients..

[196] Helsedirektoratet (Norwegian Directorate of Health). Nutrition Recommendations to Promote Public Health and Prevent Chronic Diseases. Oslo: Helsedirektoratet (Norwegian Directorate of Health); 2011.

[197] Kennedy GL, Pedro MR, Seghieri C, Nantel G, Brouwer I (2007). Dietary diversity score is a useful indicator of micronutrient intake in non-breast-feeding Filipino children. J Nutr.

[198] Vilela S, Oliveira A, Ramos E, Moreira P, Barros H, Lopes C. Association between energy-dense food consumption at 2 years of age and diet quality at 4 years of age. 2014;111(7):1275–1282. 10.1017/S0007114513003620 24229473

[199] World Health Organisation. Food and Nutrition Policy for Schools: A Tool for the Development of School Nutrition Programmes in the European Region.. In: Europe TROf, editor. Copenhagen2006.

[200] Ríos EM, Sinigaglia O, Diaz B, Campos M, Palacios C. Development of a Diet Quality Score for Infants and Toddlers and its association with weight. Journal of nutritional health & food science. 2016;4(4). 10.15226/jnhfs.2016.00171 PMC5283385 28154840

[201] Women I, and Children (WIC),. Infant Feeding Guide, A Guide for Use in the WIC and CSF Programs. Washington, DC2009.

[202] World Health Organisation. Guiding Principles for Complementary Feeding of the Breastfeed Child.. In: Pan American Health Organization, editor. Washington, DC2001.

[203] American Academy of Pediatrics. Food and Feeding 2011.

[204] Crombie IK, Kiezebrink K, Irvine L, Wrieden WL, Swanson V, Power K (2009). What maternal factors influence the diet of 2-year-old children living in deprived areas? A cross-sectional survey. Public Health Nutr..

[205] Caroline Walker Trust (1998). Eating Well for Under-5s in Care. Report of an Expert Working Group. Lomdon.

[206] Trichopoulou A, Costacou T, Bamia C, Trichopoulos D (2003). Adherence to a Mediterranean diet and survival in a Greek population. The New England journal of medicine..

[207] Mariscal-Arcas M, Velasco J, Monteagudo C, Caballero-Plasencia MA, Lorenzo-Tovar ML, Olea-Serrano F (2010). Comparison of methods to evaluate the quality of the Mediterranean diet in a large representative sample of young people in Southern Spain. Nutricion hospitalaria..

[208] Mariscal-Arcas M, Romaguera D, Rivas A, Feriche B, Pons A, Tur JA (2007). Diet quality of young people in southern Spain evaluated by a Mediterranean adaptation of the Diet Quality Index-International (DQI-I). Br J Nutr..

[209] World Health Organisation (1996). Preparation and Use of Food-Based Dietary Guidelines. Report of a Joint FAO/WHO Consultation.

[210] U.S. Department of Agriculture (USDA) (2001). Dietary Guidelines from Around the World. Food and Nutrition Information Center.

[211] INFH-CAPM (Institute of Nutrition and Food Hygiene CAoPM. The Food Consumption Tables.. House PsMP, editor. n.d.

[212] SBCNS (Standing Board of the Chinese Nutrition Society) (1999). Dietary guidelines and the food guide pagoda for Chinese residents, balanced diet, rational nutrition and health promotion.

[213] Ortega R, Lopez-Sobaler AM, Requejo AM, Andre’s P (2004). La composicio’n de los alimentos.

[214] Serra-Majem L, Ribas L, Ngo J, Ortega RM, Garcia A, Perez-Rodrigo C (2004). Food, youth and the Mediterranean diet in Spain. Development of KIDMED, Mediterranean Diet Quality Index in children and adolescents. Public Health Nutr.

[215] Serra M (2001). ¿Ma’s beneficios de la dieta mediterra’nea?. Nutricio’n y Obesidad..

[216] Monteagudo C, Palacín-Arce A, del Mar Bibiloni M, Pons A, Tur JA, Olea-Serrano F (2012). Proposal for a Breakfast Quality Index (BQI) for children and adolescents. Public Health Nutr.

[217] Arimond M, Cohen R, Dewey K, Ruel M (2005). Developing and validating simple indicators of complementary food intake and nutrient density for infants and young children in developing countries: protocol for data analysis.

[218] National Research Council (1989). Diet and Health Implications for Reducing Chronic Disease Risk.

[219] U.S Department of Health and Human Services (1988). The Surgeon General’s Report on Nutrition and Health.

[220] Chiang P-H, Wahlqvist ML, Lee M-S, Huang L-Y, Chen H-H, ST-Y H (2011). Fast-food outlets and walkability in school neighbourhoods predict fatness in boys and height in girls: a Taiwanese population study. Public Health Nutr.

[221] Lee MS, Huang LY, Chang YH, Huang STY, Yu HL, Wahlqvist ML (2012). Lower birth weight and diet in Taiwanese girls more than boys predicts learning impediments. Res Dev Disabil.

[222] U.S Department of Agriculture. Nutrition and Your Health: Dietary Guidelines for Americans. Washington, DC; 1995.

[223] Chen YC, Huang YC, Lo YTC, Wu HJ, Wahlqvist ML, Lee MS. Secular trend towards ultra-processed food consumption and expenditure compromises dietary quality among Taiwanese adolescents. Food Nutr Res. 2018;62. 10.29219/fnr.v62.1565 PMC6150927 30258346

[224] Ruel MT, Menon P (2002). Child feeding practices are associated with child nutritional status in Latin America: innovative uses of the demographic and health surveys. J Nutr..

[225] World Health Organisation. Complementary feeding of young children in developing countries. A review of current scientific knowledge. Geneva; 1998.

[226] Academy for Educational Development (AED). Recommended feeding and dietary practices to improve infant and maternal nutrition. Washington, DC; 1999.

[227] T Kennedy E, Ohls J, Carlson S, Fleming K (1995). The healthy eating index: design and applications. J Am Dietetic Assoc.

[228] Guenther PM, Casavale KO, Reedy J, Kirkpatrick SI, Hiza HA, Kuczynski KJ (2013). Update of the healthy eating index: HEI-2010. J Acad Nutr Diet.

[229] US Department of Agriculture (2020). Nutrients in 2010 USDA Food Patterns at all calorie levels. Center for Nutrition Policy and Promotion, editor.

[230] Britten P, Marcoe K, Yamini S, Davis C (2006). Development of Food Intake Patterns for the MyPyramid Food Guidance System. J Nutr Educ Behav.

[231] Guenther PM, Reedy J, Krebs-Smith SM (2008). Development of the healthy eating index-2005. J Am Diet Assoc.

[232] Marcoe K, Juan W, Yamini S, Carlson A, Britten P (2006). Development of Food Group Composites and Nutrient Profiles for the MyPyramid Food Guidance System. J Nutr Educ Behav.

[233] Kranz S, Siega-Riz AM, Herring AH (2004). Changes in diet quality of American preschoolers between 1977 and 1998. Am J Public Health..

[234] US Dept of Agriculture. The Food Guide Pyramid for Young Children 2 to 6 Years Old. In: Center for Nutrition Policy and Promotion, editor. US Dept of Agriculture; 1998.

[235] Kranz S, Hartman T, Siega-Riz AM, Herring AH (2006). A Diet Quality Index for American Preschoolers Based on Current Dietary Intake Recommendations and an Indicator of Energy Balance. J Am Diet Assoc.

[236] Marshall TA, Eichenberger Gilmore JM, Broffitt B, Stumbo PJ, Levy SM (2005). Diet Quality in Young Children Is Influenced by Beverage Consumption. J Am College Nutr.

[237] Institute of Medicine of the National Academy of Sciences (2002). Dietary Reference Intakes for energy, carbohydrate, fiber, fat, fatty acids, cholesterol, protein, and amino acids—Macronutrients.

[238] Cox DR, Skinner JD, Carruth BR, Moran Iii J, Houck KA (1997). Food Variety Index for Toddlers (VIT): Development and Application. J Am Diet Assoc.

[239] Skinner JD, Carruth BR, Houck KS, Bounds W, Morris M, Cox DR (1999). Longitudinal study of nutrient and food intakes of white preschool children aged 24 to 60 months. J Am Diet Assoc.

[240] Sharafi M, Peracchio H, Scarmo S, Huedo-Medina TB, Mayne ST, Cartmel B (2015). Preschool-Adapted Liking Survey (PALS): A brief and valid method to assess dietary quality of preschoolers. Childhood Obes.

[241] Drewnowski A (2009). Defining nutrient density: development and validation of the nutrient rich foods index. J Am College Nutr.

[242] The American Dietetic Association (2007). Practice paper of : nutrient density: meeting nutrient goals within calorie needs. J Am Diet Assoc.

[243] Chiuve SE, Fung TT, Rimm EB, Hu FB, McCullough ML, Wang M (2012). Alternative dietary indices both strongly predict risk of chronic disease. J Nutr.

[244] Harris HR, Willett WC, Vaidya RL, Michels KB (2016). Adolescent dietary patterns and premenopausal breast cancer incidence. Carcinogenesis..

[245] McCullough ML, Feskanich D, Stampfer MJ, Giovannucci EL, Rimm EB, Hu FB (2002). Diet quality and major chronic disease risk in men and women: moving toward improved dietary guidance. Am J Clin Nutr.

[246] Fung TT, McCullough M, van Dam RM, Hu FB (2007). A Prospective Study of Overall Diet Quality and Risk of Type 2 Diabetes in Women. Diabetes Care..

[247] Belin RJ, Greenland P, Allison M, Martin L, Shikany JM, Larson J (2011). Diet quality and the risk of cardiovascular disease: the Women’s Health Initiative (WHI). Am J Clin Nutr.

[248] Feskanich D, Rockett HR, Colditz GA (2004). Modifying the Healthy Eating Index to assess diet quality in children and adolescents. J Am Diet Assoc..

[249] U.S Department of Agriculture (2000). Nutrition and Your Health: Dietary Guidelines for Americans.

[250] Patterson RE, Haines PS, Popkin BM (1994). Diet Quality Index: Capturing a multidimensional behavior. J Am Diet Assoc.

[251] Kim S, Haines PS, Siega-Riz AM, Popkin BM (2003). The Diet Quality Index-International (DQI-I) provides an effective tool for cross-national comparison of diet quality as illustrated by China and the United States. J Nutr..

[252] Setayeshgar S, Maximova K, Ekwaru JP, Gray-Donald K, Henderson M, Paradis G (2017). Diet quality as measured by the Diet Quality Index–International is associated with prospective changes in body fat among Canadian children. Public Health Nutrition..

[253] Falciglia GA, Troyer AG, Couch SC (2004). Dietary Variety Increases as a Function of Time and Influences Diet Quality in Children. J Nutr Educ Behav.

[254] Martin-Calvo N, Chavarro JE, Falbe J, Hu FB, Field AE (2016). Adherence to the Mediterranean dietary pattern and BMI change among US adolescents. Int J Obes.

[255] Mediterránea. FD. Pirámide de la diéta mediterránea: un estilo de vida actual. Guía para la población adulta. 2010. Available from: http://dietamediterranea.com/piramide-dietamediterranea/.

[256] Anderson S, Kaye G, Andridge R, Smathers C, Peng J, Pirie P (2015). Interrelationships of More Healthful and Less Healthful Aspects of Diet Quality in a Low-Income Community Sample of Preschool-Aged Children. Matern Child Health J.

[257] US Department of Agriculture (2014). Health and nutrition information for preschoolers.

[258] Au LE, Gurzo K, Paolicelli C, Whaley SE, Weinfield NS, Ritchie LD (2018). Diet Quality of US Infants and Toddlers 7–24 Months Old in the WIC Infant and Toddler Feeding Practices Study-2. J Nutr.

[259] Mursu J, Steffen LM, Meyer KA, Duprez D, Jacobs DR (2013). Diet quality indexes and mortality in postmenopausal women: the Iowa Women's Health Study. Am J Clin Nutr.

[260] Hu T, Jacobs DR, Larson NI, Cutler GJ, Laska MN, Neumark-Sztainer D (2016). Higher Diet Quality in Adolescence and Dietary Improvements Are Related to Less Weight Gain During the Transition From Adolescence to Adulthood. J Pediatr.

[261] Günther AL, Liese AD, Bell RA, Dabelea D, Lawrence JM, Rodriguez BL (2009). Association between the dietary approaches to hypertension diet and hypertension in youth with diabetes mellitus. Hypertension..

[262] Materials Research Society (2019). World Bank classifications for developing countries.

[263] Centres for Disease Control and Prevention (2019). National health and nutrition examination survey.

[264] Vicente-Rodriguez G, Libersa C, Mesana MI, Béghin L, Iliescu C, Moreno Aznar LA, Dallongeville J, Gottrand F (2007). Healthy Lifestyle by Nutrition in Adolescence (HELENA). A New EU Funded Project. Société Française de Pharmacologie et de Thérapeutique..

[265] Golley RK, McNaughton SA, Hendrie GA (2015). A dietary guideline adherence score is positively associated with dietary biomarkers but not lipid profile in healthy children. The Journal of nutrition..

[266] Toffano R, Hillesheim E, Mathias M, Coelho-Landell C, Salomão R, Almada M, et al. Validation of the Brazilian Healthy Eating Index-Revised Using Biomarkers in Children and Adolescents. Nutrients. 2018;10(2). 10.3390/nu10020154 PMC5852730 29385742

[267] Aramouny E, Sacy R, Chokr I, Joudy B. Local Validation Study for NutricheQ Tool in Lebanon. J Comprehensive Pediatr. 2018. In Press.

[268] Delshad M, Beck KL, Von Hurst PR, Mugridge O, Conlon CA (2018). The validity and reliability of the Dietary Index for a Child's Eating in 2–8-year old children living in New Zealand. Maternal Child Nutr.

[269] Golley RK, Smithers LG, Mittinty MN, Emmett P, Northstone K, Lynch JW. Diet quality of U.K. infants is associated with dietary, adiposity, cardiovascular, and cognitive outcomes measured at 7–8 years of age. J Nutr. 2013;143(10):1611–7. 10.3945/jn.112.170605 23946339

[270] Hurley KM, Oberlander SE, Merry BC, Wrobleski MM, Klassen AC, Black MM (2009). The healthy eating index and youth healthy eating index are unique, nonredundant measures of diet quality among low-income, African American adolescents. J Nutr.

[271] Lazarou C, Panagiotakos DB, Spanoudis G, Matalas A-L (2011). E-KINDEX: A Dietary Screening Tool to Assess Children's Obesogenic Dietary Habits. J Am College Nutr.

[272] Moursi M, Treche S, Martin-Prevel Y, Maire B, Delpeuch F (2009). Association of a summary index of child feeding with diet quality and growth of 6–23 months children in urban Madagascar. Eur J Clin Nutr.

[273] Barnes TL, Crandell JL, Bell RA, Mayer-Davis EJ, Dabelea D, Liese AD (2013). Change in DASH diet score and cardiovascular risk factors in youth with type 1 and type 2 diabetes mellitus: The SEARCH for Diabetes in Youth Study. Nutr Diabetes..

[274] Liese AD, Bortsov A, Gunther AL, Dabelea D, Reynolds K, Standiford DA (2011). Association of DASH diet with cardiovascular risk factors in youth with diabetes mellitus: the SEARCH for Diabetes in Youth study. Circulation..

[275] Jacka FN, Kremer PJ, Berk M, de Silva-Sanigorski AM, Moodie M, Leslie ER, et al. A prospective study of diet quality and mental health in adolescents. PLoS One. 2011;6(9):e24805. 10.1371/journal.pone.0024805 PMC3177848 21957462

